# Self-assessment of the home environment to plan for successful ageing: Report from a digital health co-design workshop

**DOI:** 10.1371/journal.pdig.0000069

**Published:** 2022-07-07

**Authors:** Kate Laver, Aaron Davis, Ian Gwilt, Roslyn Dalistan, Rachel Lane, Heather Block

**Affiliations:** 1 College of Medicine and Public Health, Flinders University, Adelaide, South Australia; 2 UniSA Creative, University of South Australia, Adelaide, South Australia; University of Technology Sydney, AUSTRALIA

## Abstract

Many middle aged and older people will need to adapt or modify their home in order to age in place. Arming older people and their families with the knowledge and tools to assess their home and plan simple modifications ahead of time will decrease reliance on professional assessment. The objective of this project was to co-design a tool which enables people to assess their own home environment and make future plans for ageing in the home. We recruited members of the public who were aged 60 or older to attend a series of two co-design workshops. Thirteen participants worked through a series of discussions and activities including appraising different types of tools available and mapping what a digital health tool might look like. Participants had a good understanding of the main types of home hazards in their own homes and the types of modifications which may be useful. Participants believed the concept of the tool would be worthwhile and identified a number of features which were important including a checklist, examples of good design which was both accessible and aesthetically pleasing and links to other resources such as websites which provide advice about to make basic home improvements. Some also wanted to share the results of their assessment with family or friends. Participants highlighted that features of the neighbourhood, such as safety and proximity to shops and cafes, were also important when considering the suitability of their home for ageing in place. Findings will be used to develop a prototype for usability testing.

## Author summary

This program of work involves taking a proactive approach to educating and supporting middle and older aged people to remain in their own homes and communities for as long as possible. The aim of this project is to design a digital health tool which could be used by the public to assess the home environment and understand what steps may be needed to alter the home to improve age-friendliness. Two co-design workshops were held with older adults (aged 60–70) to understand their experience and views for such a tool. Workshop participants reported that they would use the tool to help them plan to update or modify their home as they age. The tool could also assist in decision making where it was more beneficial to move home (rather than modify). Participants raised that it was important to consider not only how one could remain at home but within the wider community environment. Usual home assessment tools tend to focus on safety and access however, participants spoke about the importance of aesthetics and pleasant spaces which supported their interests and maintaining connections with family and friends. These views will be incorporated into a prototype for usability testing.

## Introduction

Most people have a strong desire to remain in their own homes for as long as possible [1]. Middle and older aged adults report feeling strongly connected to their home and community [2]. Home is described as a place to enjoy privacy and feel free and comfortable [3]. Both the home and the objects within the home provide the sense of being surrounded by memories [4]. Furthermore, the home is considered to be the person’s base within their community which contributes to their identity and lifestyle [5]. The alternatives to remaining at home include moving to another house or to residential care either within or outside of one’s existing neighbourhood. Negative views about residential aged care are common and there is a perception that residents are lonely, lack autonomy and have poor quality of life [6].

Approximately 74% of older Australians rate their health as being good, very good or excellent [7], and most older Australians either rent or own private dwellings (99% of people aged 65–74 years and 75% of people aged 85 and over) [1]. However, as age advances, the ability to remain at home may be compromised [7] with only 66% of people aged 90–94 living in a private dwelling. Functional decline, cognitive impairment, limited social support and onset of chronic health conditions are the main predictors of nursing home admission [8–10]. Poor home environment may also trigger a move or change in housing and living arrangements. Large properties, homes that require significant maintenance, poorly designed bathrooms and flights of stairs are among design features that pose challenges to remaining in one’s home. Middle and older aged people face complex choices as to whether update or modify existing homes or move to a home which is more appropriate for ageing. Despite common perceptions, moving to a retirement village is uncommon [1]. A further consideration is whether the person may move into alternative housing but remain within their existing community [11]. Pani-Herreman et al., describe the complexity of the ‘ageing in place’ literature where the ‘place’ is not just the home but includes the person’s social networks and supports [12].

The World Health Organization is calling for improved age-friendliness of communities [13] but at an individual level there are many home modifications [14] that can be made to increase the accessibility and age-friendliness of a house including installation of grab bars, shower modifications (installation of seat and hose), removal of loose flooring (rugs) and improved lighting. These types of modifications are recommended and prescribed by an occupational therapist following a home assessment. Occupational therapists may draw on a range of existing home assessment tools to assist their practice [15,16]. Most tools have been developed for administration by a health professional, for use with older people who already have disability and the goal of most tools is to prevent falls or injury [17]. There is strong evidence from randomised trials that home assessments completed by an occupational therapist improve function in older people and reduce the rate and risk of falls among older adults [18]. However, access to occupational therapy home visits is limited; they are usually available only after injury or illness and are often limited in rural areas. Workforce shortages of allied health, particularly in aged care, further compound the problem and mean that exploration of other solutions is warranted.

This current project is part of a program of work which seeks to develop and validate digital tools so that middle and older people can take a proactive approach and assess their own homes in preparation for ageing within the home. The tool is intended to be used for future planning and therefore predominantly for use with people who do not yet have significant disability but are thinking about how they may manage at home as they age. A proactive approach to ensuring age-friendly housing may result in prevention of injury, supporting older people to stay safely at home for longer and reduced costs to the individual and society. Self-assessment methods are becoming more popular over time for several reasons. Firstly, there is good evidence that patients that are active in managing their own health and social care have better health outcomes and more positive care experiences [19]. Secondly, the public are increasingly searching online for health information. An estimated 80% of Australians report searching for health information online [20]. It is important that the public have access to tools which have credibility and validity. Thirdly, many countries are experiencing both an ageing population and workforce shortages and so solutions to manage demand are required.

Participatory research and co-design processes are being increasingly used to develop new technologies which are both useful and usable for the intended group. Involving end-users in the design process may also increase empowerment in participants [21]. Research to date has shown that participants involved in the process require information, time and a range of interactive activities in order to fully participate as designers rather than strictly as consumers of health care products [22]. Older adults, in particular, may benefit from low-tech and varied approaches to co-design [23]. This project uses a co-design process and a variety of activities to understand older adults’ perspectives on a suitable home for ageing and create a useful and usable tool. The development process differs from the development of other home assessment tools which have been developed by health care professionals with the aim of maximising safety and managing risk.

### Objectives

The aim of this program of work is to create new knowledge and digital tools for older people to assess their own home for access and safety, and arrange modifications, where required, enabling them to remain independent for as long as possible. The objective of this part of the project was to co-design a tool which enables people to assess their own home environment and make future plans for ageing in the home. Specifically, research questions included: what features a tool may include, what characteristics of self-assessment tools are desirable and undesirable and how such a tool could be implemented in practice.

## Methods

Co-design workshops were held with older people to understand their experiences and capture their views and preferences for the development of the digital tool. This research was approved by the Flinders University Human Research Ethics Committee (Project number 4523). The co-design process included involving end users in the process of design. There are various approaches to co-design including some specifically intended for digital health development and inclusion [24].

In this research, the co-design process was structured according to the British Design Council’s Double Diamond Design Process [25]. Four overlapping stages took place and included:

1. Discover: Working with community members to understand their experiences and generate a wide variety of potential inputs into the design process
2. Define: Working with community members to distil the initial contributions into a series of challenges that might be taken forward into a prototyping process
3. Develop: Using rapid prototyping tools to develop initial ideas for a digital intervention
4. Deliver: Translating the outputs from the co-design process into a series of design principles that have been taken forward into development.

Qualitative data were collected via written work and audio recordings.

### Participants

Members of the public were recruited to attend a series of two co-design workshops. Participants were eligible if they were aged 60 years or older, lived in their own home, and were relatively familiar with using the internet (defined as regularly searching for information online). All participants were fluent in English and able to provide informed consent. Individuals were excluded if they experienced significant cognitive or mobility impairment (diagnosis of dementia or mild cognitive impairment; unable to walk less than 100m without stopping; and/ or use a mobility aid to walk). Participants were recruited through social media advertising (Facebook) and contacted the research team if they were interested in participating. They were provided with a copy of a study participant information sheet and provided written consent. Participants were provided with small honorarium in recognition of the time spent actively participating in the workshop and provided with refreshments.

### Workshop format and content

Two workshops lasting approximately 2.5 hours each were scheduled two weeks apart during 2021. The sessions were led by a facilitator and academic with extensive experience in facilitation and co-design (AD, PhD, male), the lead researcher (KL, PhD, female) and support staff and held in a large meeting room within the University. Participants were not previously known to facilitators (AD or KL). Over the course of the workshops, participants were introduced to the facilitators and their previous work, provided with information about the objectives of the research project and the research funding, informed about the content of the co-design session, then led through a series of interactive activities. Copies of the workshop activities and outputs are available as supplementary files.

#### Workshop 1 (Discover and Define)

The first workshop began with the facilitators providing an introduction to the study and its purpose. Each participant was then given a piece of paper printed with two conversation starters and areas to write down their thoughts: 1) imagining yourself at 85 or 95, what does a perfect home allow you to do? And 2) some homes are built in more challenging ways to keep people fit as they age. How can a home keep you fit and healthy? After individually documenting, then sharing their thoughts with the larger group, participants were divided into four small groups for card sort activities. Each group was provided a template, titled: What is most important about home? and a range of images from which they had to select images that resonated with them. Images were pasted onto the template along with a written explanation of what the image represented and why. Finally, each participant was handed a second template titled: Things I think will need to be adapted as I get older—and cards for features such as ‘bedroom’, or ‘slippery flooring’. Participants were asked to identify areas of the home that may require modification to enable successful ageing and to rate the difficulty in making these changes. In between activities, individual and group sharing occurred which was audio recorded.

#### Workshop 2 (Develop)

The second workshop began with a recap of the work conducted so far followed by an activity in which groups appraised existing self-assessment tools. Four small groups were given a range of self-assessment tools from different fields (for example, assessment of insurance needs, assessment of heart health) and in different mediums (for example, website, printed form). Using sticky notes, they commented on features of the tool that worked (e.g., easy to read) and those that didn’t (e.g., out of date information). Participants were then asked to individually design what a digital health tool might look like in terms of feature and content. They were provided with a template and pictures to promote thinking about what features should be included and how they might work together. Finally, participants worked individually on a storyboarding exercise where they mapped who, what, when, why and how the tool could be used (‘day in the life’ activity). Group sharing time was audio recorded. The facilitator used their groupwork skills to encourage equal participation and open-mindedness. Participants were able to talk freely in the activities and were notified when the audio recording occurred during the whole group sharing activities.

### Analysis of results

All project materials (written materials, pictures, audio recordings) were collated. Data for each activity were extracted into an Excel spreadsheet in preparation for content analysis. Qualitative data (such as written responses to the conversation starters) were analysed by coding and categorising similar responses. For example, ‘safety’, ‘feel safe’ and ‘safety-in and out of the house’ were grouped under the theme of ‘feeling safe’. This process of coding and categorising was completed by one researcher (KL) and checked by another (RL). Instances of disagreement were resolved through discussion or discussed with a third author (HB) who assisted in resolving differing opinions. The range of views was reported with consideration of frequent responses and all codes were derived from the data (rather than being identified in advance). The final themes and categories were discussed by the team prior to finalisation. Audio recorded content was reviewed by KL and RL to check whether it generated any additional data not captured on the written materials. Data from the first workshop was shared-back and the interpretation checked with participants at the start of the second workshop.

## Results

Fifteen members of the public (nine women and six men) aged over 60 were recruited to participate and attend the two workshops. Of the 15 recruited, two were unable to attend workshops due to illness, and one was only able to attend one workshop due to its location. Attendance therefore was 12 participants in the first workshop and the same participants along with one additional participant (n = 13) in the second workshop.

### Discover and define (Workshop 1)

Key priorities in older age (85–95 years) identified by the group in the opening conversation were staying independent, maintaining health and feeling safe. Participants also prioritised participation in leisure activities and seeing friends and family. Living in an accessible environment was identified as a priority as was being in a pleasant environment (e.g., “surrounded by green—view” and “access to outdoors”).

A range of different ideas were raised as to how a home can keep you fit and healthy. Being accessible was important (“level surface”) as was being free of clutter. Participants valued the surrounds (“visually pleasant–sunshine, windows”) and linked pleasant surroundings to higher mood (“outlook inspires and lifts my spirits”). Being connected to the community was important (“proximity to public transport and parks”) and some participants spoke of the need for good access to technology and the internet.

Small groups compiled images and descriptions of what they felt was important about a home *(See S1 Image).* Pleasant surroundings which were warm and light were identified as important. Groups discussed wanting to feel safe, supported and free and to be connected to others (both in person and through use of technology). The home was also viewed as a place to support leisure pursuits (“learning and relaxation”, “art and creativity”). A key concept identified by the group was how spaces within the home could be adapted over time and as needs changed.

There were many common responses when participants were asked about what aspects of their own homes would have to be modified as they aged. Participants appeared to have reasonable knowledge about the types of home hazards that may exist and some possible solutions. Common responses included requiring handrails and wider doorways. Bathrooms and gardens were highlighted as being the areas of the house which would require the most attention. Our participants raised the idea of the home being a space which could be adapted over time to support changing needs.

### Develop and deliver (Workshop 2)

#### Appraisal of existing tools

Three small groups appraised existing self-assessment tools to identify helpful and non-helpful features. Positive attributes identified included simple checklists and language that was easy to understand but that didn’t “talk down” to the audience. Presenting the checklist as questions for the reader (with answers) was viewed as being more engaging and inviting participation in contrast to fact sheet type information. Websites that contained visually appealing images and were easy to navigate (“so you didn’t go round in circles”) were highly regarded. Negative attributes included small and dense text including too much jargon. Sites which looked “home made” and lacked visual appeal (“lack of contrast”, “images too small”) were poorly received. Information which was patronising or adopted a “fearful” tone were also unpopular. Participants also were sceptical of credibility of information (“what does 300^th^ most walkable neighbourhood mean?”, “Evidence–from where?”).

#### What the app might look like

Common features were grouped into categories. Categories and examples are presented in Table 1.

**Table 1:** Desirable features of a home assessment application.

Feature	Example
**Assessment**	Checklist of features to improve accessibility and age-friendliness of the home. For example, ‘can controls for heating and air-conditioning be easily accessed?’
**Occupational therapy advisor**	An audio walkthrough of a home pointing out features to be aware of
**Calculators**	To enable calculation of costs of different types of modifications
**Information and inspiration**	Articles, resources, information about trades and products. Tips and tricks for home modifications
**Interaction**	Chatroom, ability to share information with others
**Community**	Ability to find out information about local neighbourhood, eg. local transport options
**Personalisation features**	Ability to create personal profile and settings, work in progress folder with ability to save information and keep progressive notes

#### Implementation considerations

Participants identified friends, local advocacy groups for older people (i.e., Council of the Aged), local councils, newsletters, social media, mainstream media, general practitioners and government websites as possible sources of dissemination for the digital health tool. Being available on a website or their mobile phone’s App Store were the most likely sources of access. They believed they would use it if they were interested in the topic or had been recommended use by a family member or friend. This may have also been triggered by a change in functional ability. Participants identified that it would be helpful to have some sort of record stemming from the self-assessment which they could use as an action plan and could use this to work from as time and budget allowed. They also felt it would be useful to being able to amend their assessment over time.

## Discussion

The data generated from the workshops enabled the development of a blueprint for a digital tool for self-assessment of the home environment. The co-design workshops revealed that participants had good insights into the effects of ageing and how this might impact on their ability to live within their own home. They were able to identify what was important to them about ‘home’ and had ideas about what would be needed in order to modify their own homes. The two workshops provided participants with the time and information needed as well as varied activities to enable the participant to think as a designer.

Our research questions included wanting to understand what features a home self-assessment tool may include and a clear idea of important features was established (as presented in Table 1). The data revealed which characteristics of self-assessment tools were desirable and undesirable and participants reported that clarity, credibility, tone of language, easy navigation and visual appeal were important. In preparation for implementation, it was important to understand how such a tool could be implemented in practice and participants suggested dissemination through a range of avenues including media.

Data from the ‘day in the life’ activity suggested that most participants believed that a digital tool would be useful in developing an action plan for improving the age-friendliness of their homes, and that it could increase the scale and reach of existing home safety programs. In particular, the digital approach was as acknowledged as being an opportunity to provide advice and guidance before a critical event triggered the need for immediate action. This shift from reactive to proactive engagement in home modification was implicit in the rationale for the project but not specifically raised with participants which makes its emergence through the co-design process a positive affirmation of the digital assessment approach.

Participants reported the need to consider many features of the home which are currently addressed in home assessment checklists designed for use by health professionals. Current home visit checklists tend to focus primarily on safety and access. Existing tools frequently consider safety features such as handrails and slippery floors [15,26]. It was clear from the participants that while these were important there were many other features of the home that contribute to comfort. There is less often discussion about the home as a space for being creative and to pursue leisure activities. Our participants suggested adaptations to the home and spaces over time are necessary yet changes in home layout and use of spaces are seldom considered in practice. Participants also frequently discussed the need for home to be a pleasant and comfortable environment with access to outdoors, fresh air, views, warmth and light (as seen in the data provided in the appendices). These features of a home were discussed as being desirable but are not currently addressed in home assessment tools used by occupational therapists in clinical practice [15–17].

The provision of spaces for leisure activities while also keeping the home “free of clutter and obstacles” presents an interesting insight into the challenge of downsizing or moving to a retirement village. In contrast to the belief that as people age “extra bedrooms” are no longer necessary, the use of these spaces as leisure and creative spaces may be underestimated. Similar findings have been found in other recent research [27] with social, creative and cultural activities highlighted as important uses of “extra bedrooms”.

Another issue raised by participants was not just their ability to age successfully within the home but within the community. Current approaches tend to assume that older people are relatively immobile and rarely leave the home. In contrast, data shows that many older people are active in their communities with 87% visiting people outside their home and 28% going on holiday with others in the last 3 months [7]. These findings demonstrate the importance of considering the person’s community environment in addition to their home environment. Maintaining social and community participation as much as possible over time are associated with successful ageing and both individual and community efforts are required to achieve this outcome [28,29]. It is also possible that the purpose of a home assessment may be to help the person come to the realisation that the current home or community is not suitable for ageing and that moving to another dwelling within their community or to another community may be more appropriate than pursuing home modifications. It may be appropriate to stimulate these conversations at a time when people have the financial, emotional and physical resources to move (earlier in the ageing journey).

At the conclusion of the workshop, the desirable features and characteristics of a digital health tool for home self-assessment were apparent. The next step in this research is to develop the prototype and test the usability of the digital health tool with the intended population and refine the tool as needed. Finally, we will explore levels of agreement between self-assessment (conducted by a middle or older aged member of the public) and an occupational therapist will be explored.

A number of limitations in this co-design project should be acknowledged. Recruitment of participants via social media (Facebook) likely resulted in a lack of diversity excluding those from lower sociodemographic backgrounds and with lower levels of digital literacy. However, the profile of the end users of the digital health tool is likely similar to those who use Facebook. The workshops did not include people with disabilities significantly affecting their mobility as their access needs and therefore modifications required are likely to be much more complex requiring occupational therapy assessment and input. Work is required to consider alternative solutions for other populations.

## Conclusion

In conclusion, supporting older people to remain independent in their own homes and communities is a mutual goal of older people, policy makers and governments. This co-design process affirmed that a digital tool would be acceptable and useful in assisting middle and older people with decision making. Participants identified important aspects of ageing in place beyond standard home modifications which will be incorporated by the researchers in the next phase of this research.

## Supporting information

S1 Image Card sort activity. (TIF)

S1 Appendix Workshop 1 activity data. (PDF)

S2 Appendix Workshop 2 activity data. (PDF)

S3 Appendix Workshop transcript. (PDF)
