# Supplementary figures and images for: Self-assessment of the home environment to plan for successful ageing: Report from a digital health co-design workshop
Source: PLOS Digit Health. 2022 Jul 7;1(7):e0000069. doi: 10.1371/journal.pdig.0000069 (PMC9931232; doi:10.1371/journal.pdig.0000069)

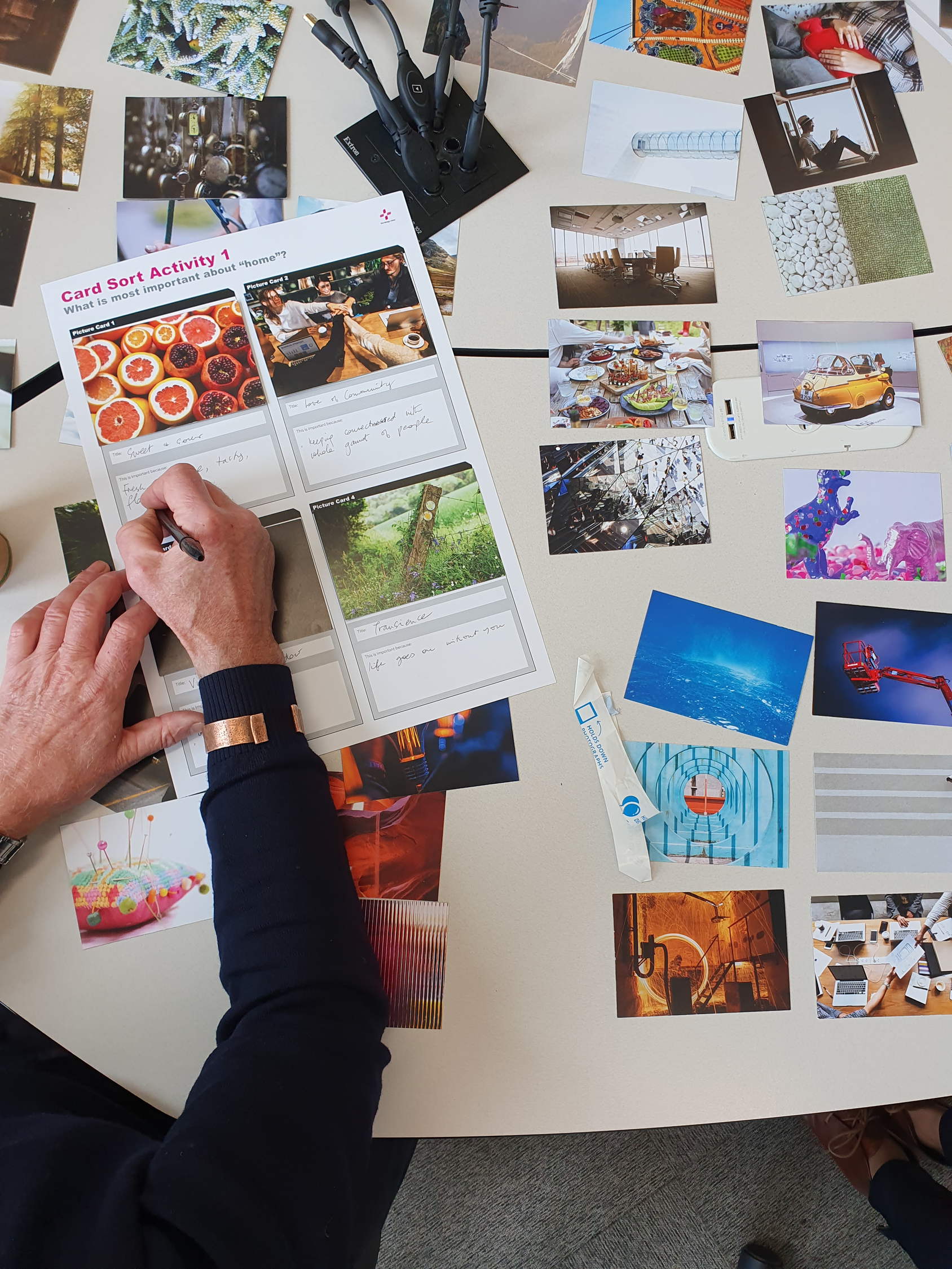

Supplement: S1 Image — (TIF) [file pdig.0000069.s001.tif]
