# Supplementary material for: Self-assessment of the home environment to plan for successful ageing: Report from a digital health co-design workshop
Source: PLOS Digit Health. 2022 Jul 7;1(7):e0000069. doi: 10.1371/journal.pdig.0000069 (PMC9931232; doi:10.1371/journal.pdig.0000069)
Supplement: S1 Appendix — (PDF) [file pdig.0000069.s002.pdf]

# Conversation Prompt 1

Project funding:

Australian Government
Australian Research Council

Project partners:

Flinders University

University of South Australia

the design clinic

**Many people wish to remain in their own homes for as long as possible.**

**Imagining yourself at 85 or at 95, what does a perfect home allow you to do?**

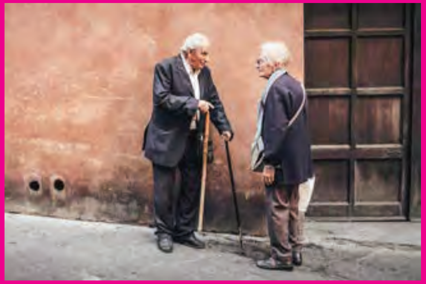

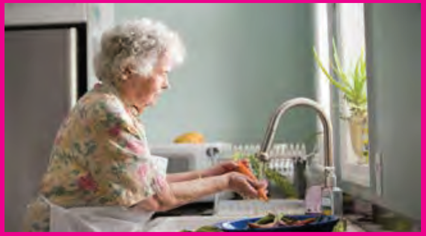

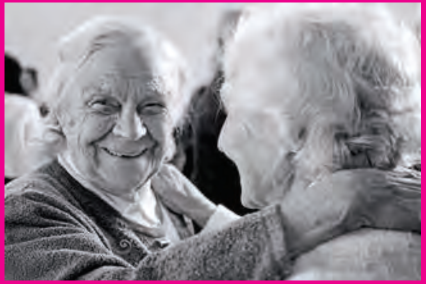

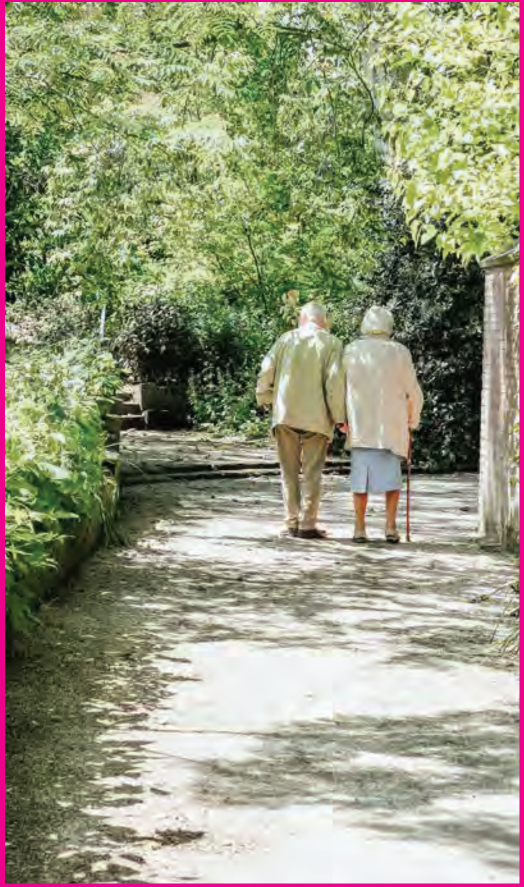

## Imagining yourself at 85 or at 95, what does a perfect home allow you to do?

**Priorities at age 85 or 95:**

- mobile - be
- Faculties - sight
- surrounded by green - view

→ safety → house
→ locality

→ accessible to friends

- garden - small

**Ways a home can support this:**

→ level surface

→ outlook inspires and lifts my spirit

→ visually pleasant
- sunshine
- windows

→ insulated

## Imagining yourself at 85 or at 95, what does a perfect home allow you to do?

### Priorities at age 85 or 95:

Reading
Cooking for family & friends
Good health
Walking
travel
Garden small
Safety

### Ways a home can support this:

good lighting
wall oven - electric hot plates that turn off after no use.
apartment living gives a sense of community life
Being able to lock up and leave easily
Courtyard garden - Pots Plants

## Imagining yourself at 85 or at 95, what does a perfect home allow you to do?

**Priorities at age 85 or 95:**

Have long lunches.
Feel lifted in the morning
Feel safe. –
Be with friends
~~Keep~~ No need to get rid of everything.
Be reasonably independant.
Be comfortable. Warm - Cool
Go for walks.

**Ways a home can support this:**

Hand rails.
Regular cleaner
An outside space with sunshine.
Double glazing
Raised chairs etc
Close neighbourhood.

## Imagining yourself at 85 or at 95, what does a perfect home allow you to do?

### Priorities at age 85 or 95:

- TO CONTINUE AN INTELLECTUAL LIFE. I VALUE LEARNING + KNOWLEDGE.
- ALSO, TO CONTINUE WALKING. SOLITARY WALKING IS MY EXERCISE + MEDITATION... IN THE BUSH + HILLS IF POSSIBLE.
- FINALLY, TO RETAIN CONTROL OVER MY AESTHETIC CHOICES... TO NOT HAVE OTHERS' NOISE OR MUSIC IMPOSED ON ME (OR MINE ON THEM)

### Ways a home can support this:

- ON-LINE ACCESS. I.T. WITH A GOOD KEYBOARD
- PROXIMITY TO PUBLIC TRANSPORT + PARKS
- EXTERNAL + INTERNAL SOUND ISOLATION. PRIVATE SPACE I CAN ARRANGE.

## Imagining yourself at 85 or at 95, what does a perfect home allow you to do?

| **Priorities at age 85 or 95:** | **Ways a home can support this:** |
| --- | --- |
| Access to outdoors<br>connect with nature<br><br>Storage that is accessible<br>& safe to access<br><br>communication – age friendly ~~[illegible]~~ IT<br><br>community access to all<br>groups & ages. | single storey<br>not cluttered<br>min. steps |

## Imagining yourself at 85 or at 95, what does a perfect home allow you to do?

### Priorities at age 85 or 95:

- ACCESS
- OVERHEAD CUPBOARDS – [illegible] CUPBOARD
- LIVING INDEPENDENTLY.
- [illegible] LIGHT FITTINGS OR ELECTRONICALLY SELF SUFFICIENT
- COUNCIL REPRESENTATION
- ACCESSING INTERNET
- COMPUTER LITERACY
- VERMIN PROOFING
- LOSING INDEPENDENCE
- TRAPPED IN HOUSE
- WICKING BEDS – GARDEN

### Ways a home can support this:

- HOME LIKE JETSONS BUT LIKE FLINTSTONES.
- [illegible] GARDEN – VEGIES ETC – FOR EXERCISE WICKING BEDS ETC

**Imagining yourself at 85 or at 95, what does a perfect home allow you to do?**

**Priorities at age 85 or 95:**

Being able to be outdoors in nature, e.g. garden

Being able to move freely around the home

Being able to access items in storage

Access to yoga and wellbeing activities - online

Feeling safe to do things for myself

Low maintenance house & garden

Communication & community access

Able to get food delivered

**Ways a home can support this:**

easy access, low maintenance garden with raised beds, insect & bird attracting plants.

User friendly fixtures & fittings

Quality, easy to use taps, appliances

Access to internet, phone, transport & deliveries

Beautiful, light-filled easy care home

Reduce clutter - live simply (but comfortable).

## Imagining yourself at 85 or at 95, what does a perfect home allow you to do?

**Priorities at age 85 or 95:**

Safety

Mobility

Access

Communication

Community

Being trapped in a horrible environment

**Ways a home can support this:**

**Imagining yourself at 85 or at 95, what does a perfect home allow you to do?**

**Priorities at age 85 or 95:**

- Being able to move from room to room.
- Things accessible (not needing to reach up)
- Accessible bathroom + toilet.
- Access to outdoor.
- Safety - in + out of house.
-

**Ways a home can support this:**

-

## Imagining yourself at 85 or at 95, what does a perfect home allow you to do?

### Priorities at age 85 or 95:

Moving from room to room easily

Not slippery

One level

Functionality

Able to do whatever you do now

then but safely.

Kitchens / bathrooms

access to outdoor spaces / view

Have visitors in line c̄ needs

Ability to close off open spaces.

### Ways a home can support this:

## Imagining yourself at 85 or at 95, what does a perfect home allow you to do?

| Priorities at age 85 or 95: | Ways a home can support this: |
| --- | --- |
| - FRESH AIR<br>- light.<br>- safe surfaces<br>- storage (safety)<br>-<br>movable<br>- reconfigurable spaces | open plan + Break.<br>change for times of the day... |

**Imagining yourself at 85 or at 95, what does a perfect home allow you to do?**

**Priorities at age 85 or 95:**

Easy and safe mobility within home.

Temperature control - warmth + cooling

Light + airy.

Accessible storage.

-> door width.
- shower/bathroom / toilet,

Small garden. - low maintenance.

**Ways a home can support this:**

**Imagining yourself at 85 or at 95, what does a perfect home allow you to do?**

**Priorities at age 85 or 95:**

easy moving around safely
not slippery
new design for bathrooms
warmth in winter
light, air, access to outdoor
adaptable space
low maintenance, easy care

**Ways a home can support this:**

no steps, sharp edges
non slippery floors
open-plan

# A perfect home when I'm 85 or 95 allows me to...

## *Priorities:*

### *Location*
- *Neighbourhood that facilitates walking. To continue walking in the bush / hills if possible*
- *Be surrounded by nature, have access to nature (e.g. a small garden) and views of nature. Fresh air, light*

### *Physical needs*
- *Maintain good health*
- *Being able to move freely around the home. Wide doors etc.*
- *Stay mobile and active. Be reasonably independant.*

### *Possessions*
- *Not force me to get rid of everything*
- *Access everything - Storage that is accessible and safe to access.*

### *Quality of space*
- *Be comfortable - warm in winter, cool in summer*
- *Maintain a positive and stimulating environment, retaining control over aesthetic choices, feeling lifted in the mornings. Not being trapped in a horrible environment*
- *Space for reading and to continue learning and building knowledge*

### *Social*
- *Be accessible to friends. Space to spend time with friends*
- *Access to all groups & ages in the local community*

### *Food*
- *Get food delivered*
- *Cooking for family & friends. Have long lunches*

### *Technology*
- *Communicate through age-friendly IT, including access to online wellbeing / community activities*

### *Safety*
- *Adapt my environment to suit my needs*
- *Live safely inside and outside of the house (house + garden + neighbourhood). Feeling safe to do things for myself. Still be able to do what I do now safely*

### *Lifestyle*
- *Travel*

## *Ways this can be supported:*

### *Location*
- *A good neighbourhood - proximity to public transport & parks*
- *Apartments can give a sense of communal life*
- *Outlook that inspires and lifts spirits. Visually pleasant (sunshine / windows etc.)*
- *A courtyard garden or easy access, low maintenance garden with raised beds, insect and bird attracting plants*

### *Quality of space*
- *Light-filled home / an outside space with sunshine*
- *A well insulated home, e.g. double glazing for energy as well as sound*

### *Lifestyle*
- *Security, including being able to lock-up and leave easily.*
- *Low maintance and easy care - inside and out*

### *Services*
- *Bringing in services to assist with some tasks, e.g. regular cleaner*
- *Facilitate easy deliveries*

### *Technology*
- *Appliances that automatically switch off*
- *Good lighting*
- *Access to the internet and other technology*

### *Safety*
- *Hand rails, no steps, sharp edges or slippery floors. No clutter - live simply but comfortably. Accessible bathroom and toilet*
- *User friendly fixtures and fittings - e.g. high-quality, easy to use taps and appliances*
- *Level surfaces and single storey home*

Project funding:

Australian Government
Australian Research Council

Project partners:

Flinders UNIVERSITY

University of South Australia

the design clinic

# Conversation Prompt 2

## Some homes are built in more challenging ways to keep people fit as they age . . .

## How can a home keep you fit and healthy?

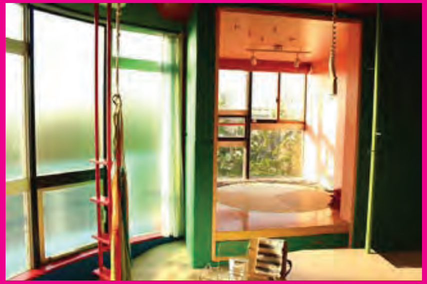

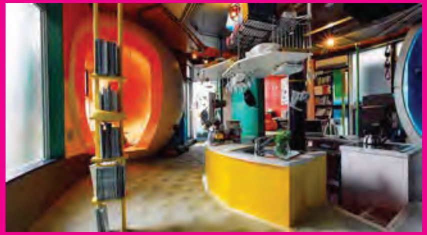

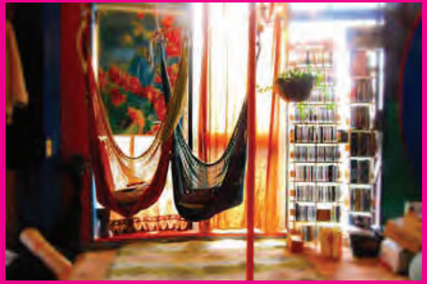

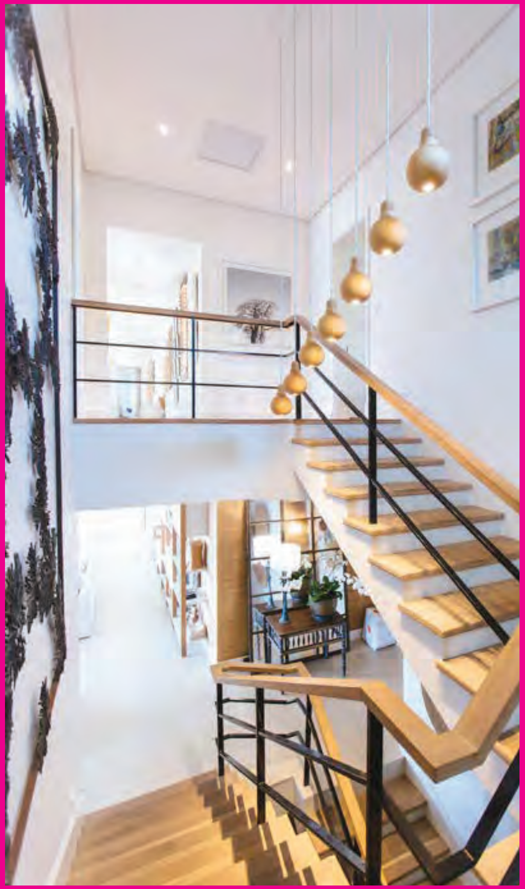

## How can a home keep you fit and healthy?

### Opportunities (Reasons For):

Kitchen that encourages movement.

Garden

### Challenges (Reasons Against):

Steps.

= Money =

## How can a home keep you fit and healthy?

### Opportunities (Reasons For):

Maintenance - Satisfaction of fixing and maintaining items

Cleaning - very good for weight control

Stairs - once again fitness in the home but also can be unsafe

Local area needs tobe safe and green

Safety

### Challenges (Reasons Against):

Stairs - tripping

Showers - not over baths

Mowing off lawns

Mainting gutters

## How can a home keep you fit and healthy?

**Opportunities (Reasons For):**

- Cleaning
- Walking spaces nearby
- neighbourhood walking
- garden —

**Challenges (Reasons Against):**

→ Physical energy

-

→ capacity

→ money → costs.

## How can a home keep you fit and healthy?

### Opportunities (Reasons For):

- LAYOUT REQUIRING MOVEMENT, NOT MINIMISING IT.
- ACCESS TO FRESH AIR / OUTDOOR ACTIVITY (NOT SITTING)
  ... A WALKABLE NEIGHBOURHOOD.
- LIGHT.

### Challenges (Reasons Against):

- STEPS + STAIRS
- HIGH CUPBOARDS
- AWKWARD LAYOUT
  (HINGING OF DOORS)
  - ALLOWANCE FOR UNOBSTRUCTED PASSAGE

## How can a home keep you fit and healthy?

### Opportunities (Reasons For):

garden - look at
- work in
- dog

weeding therapeutic

accen to music

storage with easy accen to bikes, surfboards

Location - accen to service

### Challenges (Reasons Against):

garden " Can become physically challenging.

Love where you live & don't want to move.

money

## How can a home keep you fit and healthy?

### Opportunities (Reasons For):

+ LOCATION – opportunities to walk, inviting outdoor spaces. + COMMUNITY

* 'Use it or lose it' – opportunities to move, bend, squat & stretch while doing daily chores.

* Music — so you can dance, do yoga, move whenever & wherever you are.

* A garden – another motivator to keep active.

* Stairs – motivation to keep legs & hips mobile & healthy.

Animals – eg dog to walk.

### Challenges (Reasons Against):

If something happens. You also need to have things in easy reach & easy to manage. So how can we have both?

Flexibility.

Adaptable house – can reassign rooms.

If upstairs/downstairs, can live either ↑ or ↓.

Homes built to be adaptable from the start.

## How can a home keep you fit and healthy?

### Opportunities (Reasons For):

Visually } stimulating
Auditory }

Garden - motivating

Some sort of flexability to move furniture etc.

### Challenges (Reasons Against):

Having your choices taken away from you!

## How can a home keep you fit and healthy?

### Opportunities (Reasons For):

sensory stimulation

### Challenges (Reasons Against):

fitter healthier

## How can a home keep you fit and healthy?

**Opportunities (Reasons For):**

- Room for Exercise
- Access to outdoor area,
- lighting.
-

**Challenges (Reasons Against):**

## How can a home keep you fit and healthy?

### Opportunities (Reasons For):

pets
access to outside

### Challenges (Reasons Against):

No 1 size fits all – comorbidities
– adaptability for individuals

tes ier

**How can a home keep you fit and healthy?**

**Opportunities (Reasons For):**

space(s) for exercise - inside or out.

For exercising eg walking trails in the neighbourhood.

**Challenges (Reasons Against):**

No one size fits all. - need flexibility & adaptability around changing needs of person.

## How can a home keep you fit and healthy?

### Opportunities (Reasons For):

SPACE to do SOME
EXCERSISE ....

### Challenges (Reasons Against):

NOT ONE size fits all.

- stimulating.
- (ADAPTABLE - visuality)
controll.

<- services ->

# How can a home keep you fit(er) and health(y/ier)?

## Opportunities:

*Flexibility*

*Furniture and layouts that allow cleaning and chores to be done safely as a form of exercise. Opportunities to move, bend, squat, stretch etc.*

*Flexible layouts to suit current and future needs*

*Room layouts that encourage movement rather than minimising it - e.g. kitchen*

*Physical activity*

*Neighbourhood*

*An attractive, walkable local area with designated opportunities for physical activity*

*Removing barriers*

*Storage with easy access to bikes / surfboards etc.*

*(Safe) stairs. Stairs - motivation to keep legs and hips mobile and healthy*

*Safety*

*Outdoor environments*

*A garden that is manegable at various levels of physical ability*

*Access to outdoor spaces*

*Spaces for exercise inside and outside*

*Animals - e.g. having a dog to walk*

*Supported challenges*

*Maintenance that can be done safely as you age*

*Plan for and facilitate support services coming into the home to assist as capacity decreases*

*Access to sensory stimulation - e.g. music, views, soundscapes etc.*

*Stimulation*

## Challenges:

*Trading off between loving a current home and knowing it might not be the best thing for you.*

*Akrasia*

*Cost*

*Base-layout of the home can restrict these opportunities*

*Changes can be expensive*

*Bespoke solutions*

*There is no one-size-fits-all. Any approach like this must be tailored to the individual and flexible as needs change.*

*Safety*

*Steps and stairs can be very dangerous*

*Safety: Some maintenance/ gardening/ physical activities can lead to accidents and injuries*

*Rapid shifts*

*When sudden changes happen, choice is often taken away. Adaptation must be very fast.*

*Challenges that keep you fit can very quickly become unmanageable - e.g. high-cupboards. Need a plan to address this if/when it happens.*

*As we age, energy levels tend to drop as does the capacity to do physical tasks.*

*Gardens can be very phsyically challenging*

*Physical demands*

# Card Sort Activity 1

**Card deck to be used for Card Sort 1:**

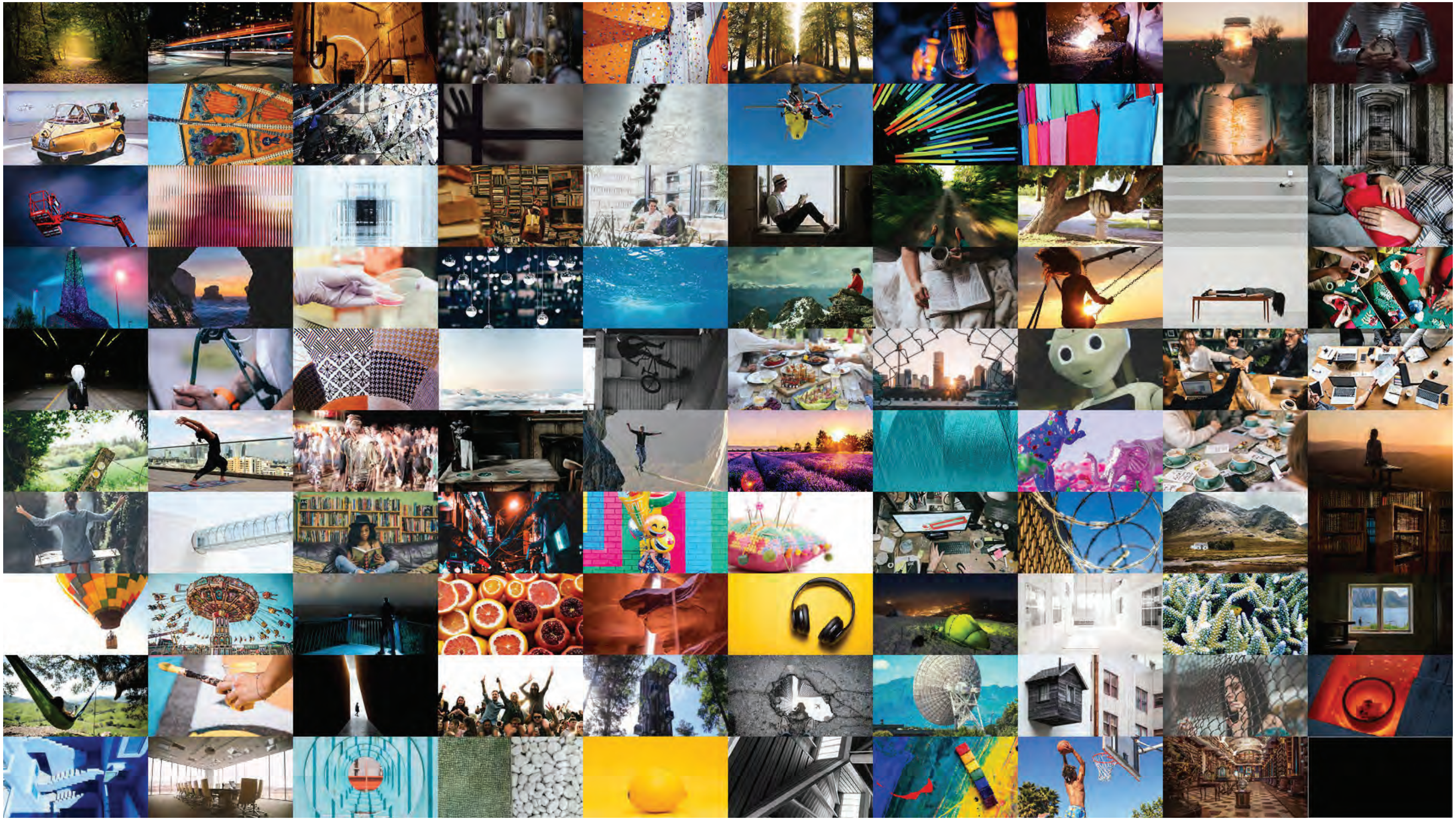

# Card Sort Activity 1

## What is most important about "home"?

the design clinic

**Picture Card 1**

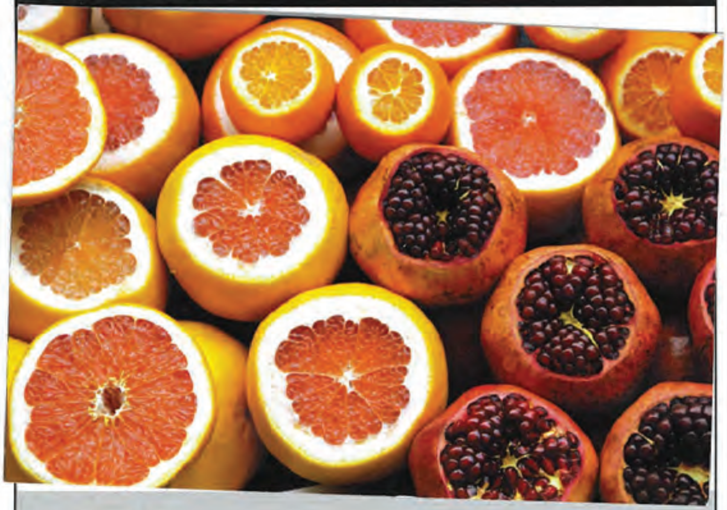

Title: Sweet & Sour

This is important because:

Fresh, colourful, tasty, flavourful, diverse.

**Picture Card 2**

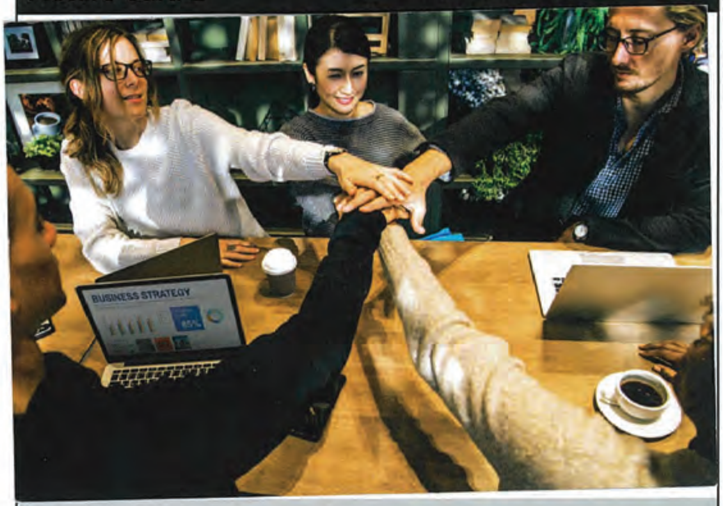



Title: Love of Community

This is important because:

- keeping connected with whole gamut of people
- Loneliness kills

**Picture Card 3**

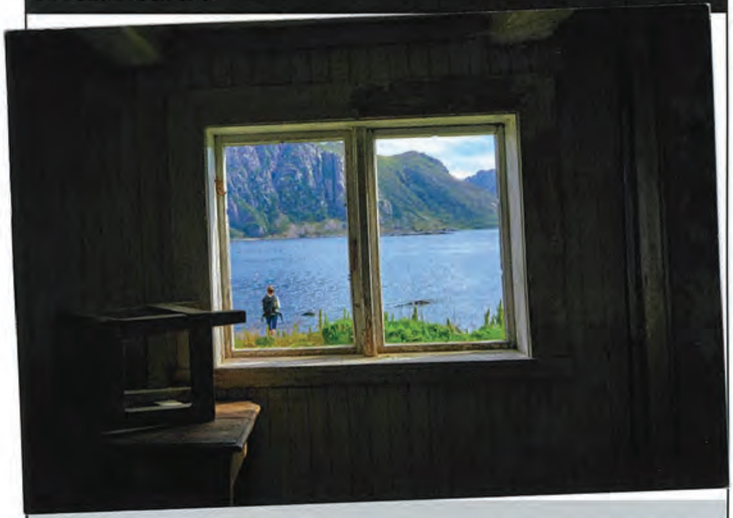

Title: View from my window

This is important because:

- sense of space
- the vista
- Importance of not being hemmed in.

**Picture Card 4**

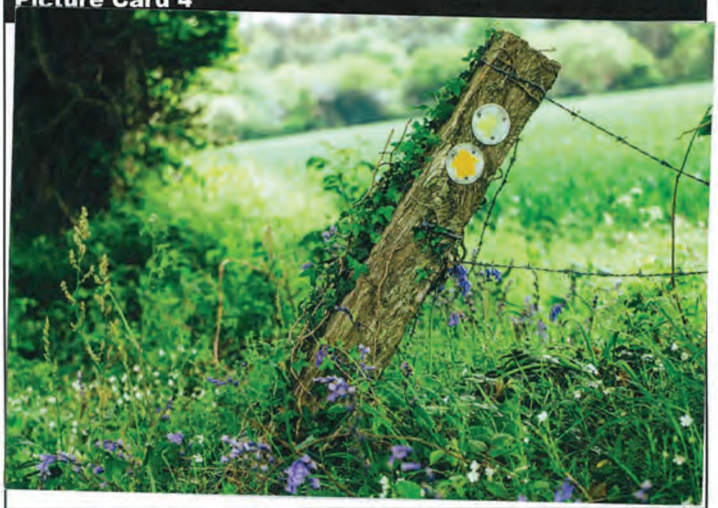

Title: Transience

This is important because:

- life goes on without you
- in the end nothing will matter

# Card Sort Activity 1

## What is most important about "home"?

the design clinic

### Picture Card 1

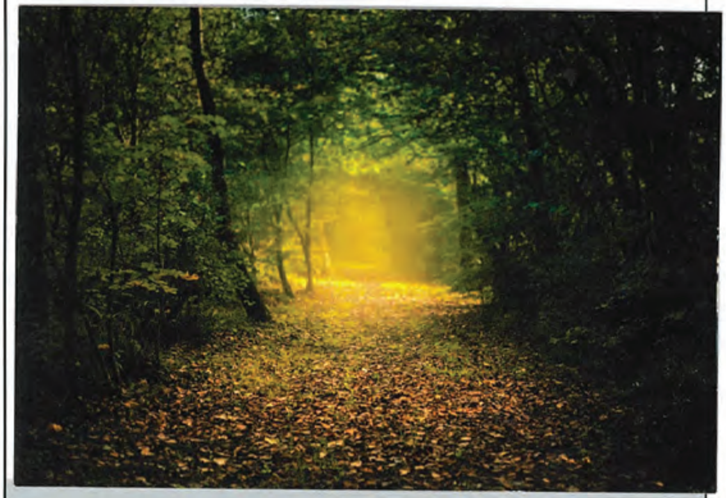

Title: ACCESS TO NATURE + LIGHT

This is important because:

Emotional wellbeing.

### Picture Card 2

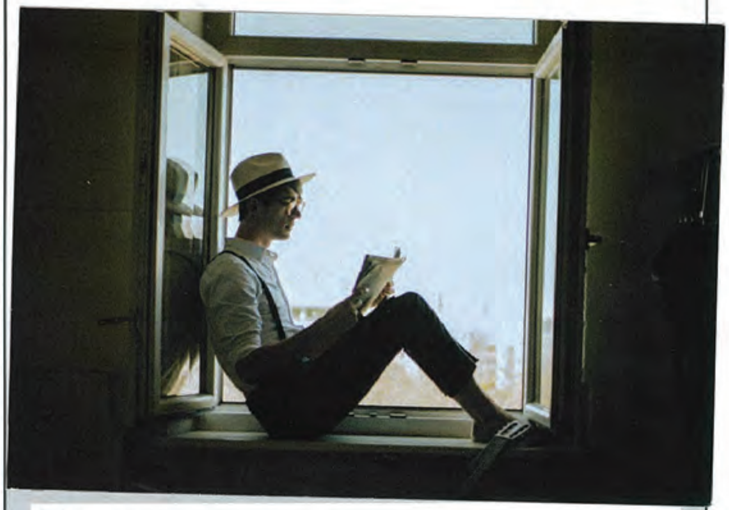

Title: ADAPTABLE SPACES

This is important because:

our needs can change over time

### Picture Card 3

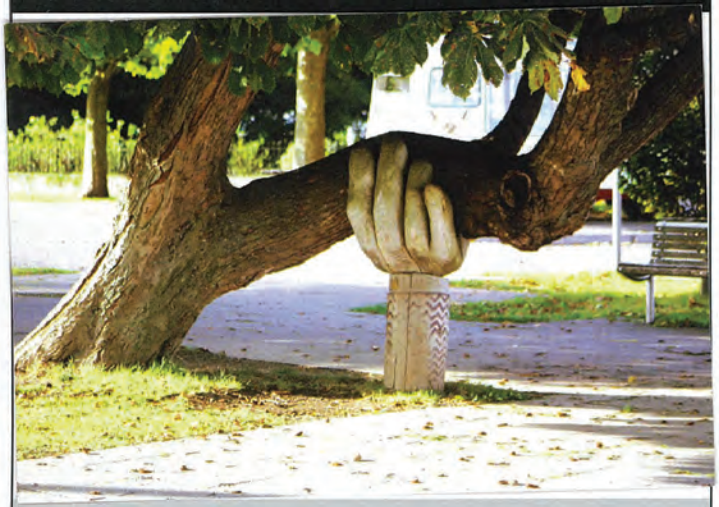

Title: SUPPORT

This is important because:

### Picture Card 4

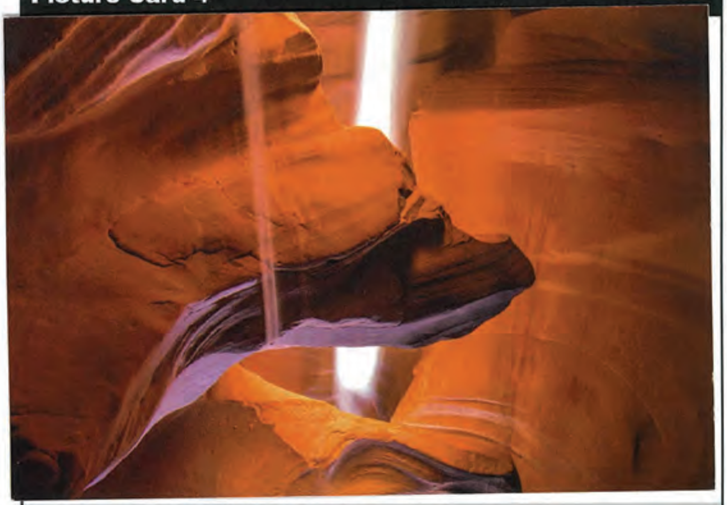

Title: INSPIRATION

This is important because:

# Card Sort Activity 1

## What is most important about "home"?

**Picture Card 1**

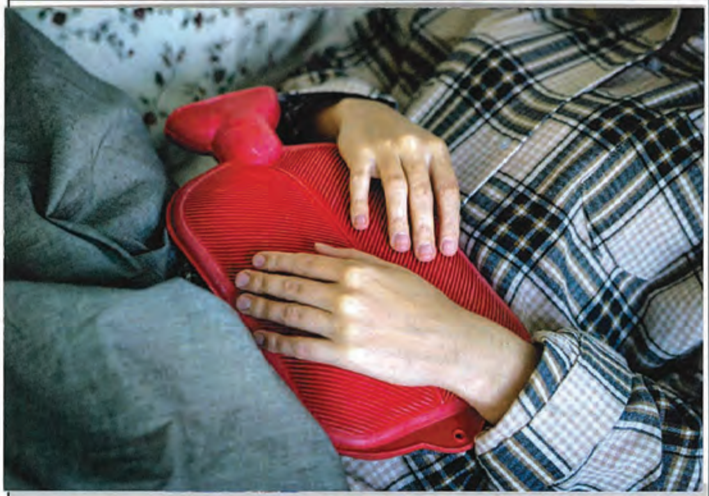

Title: SECURITY + WARMTH

This is important because:
Everyone needs it.
Emotional, psychological well being

**Picture Card 2**

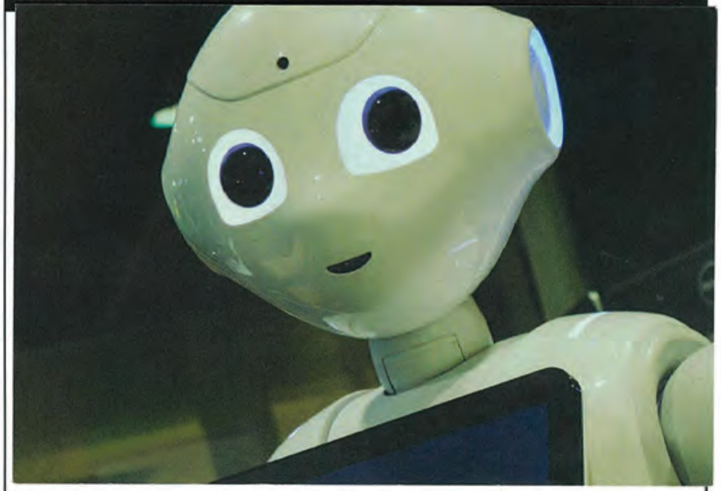

Title: TECHNOLOGY

This is important because:
The way of the future.

**Picture Card 3**

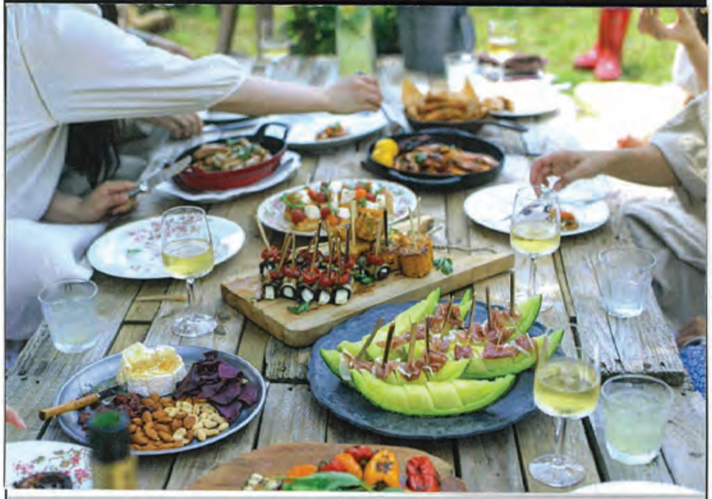

Title: FAMILY + COMMUNITY

This is important because:
Need ability to share with others, + be involved.

**Picture Card 4**

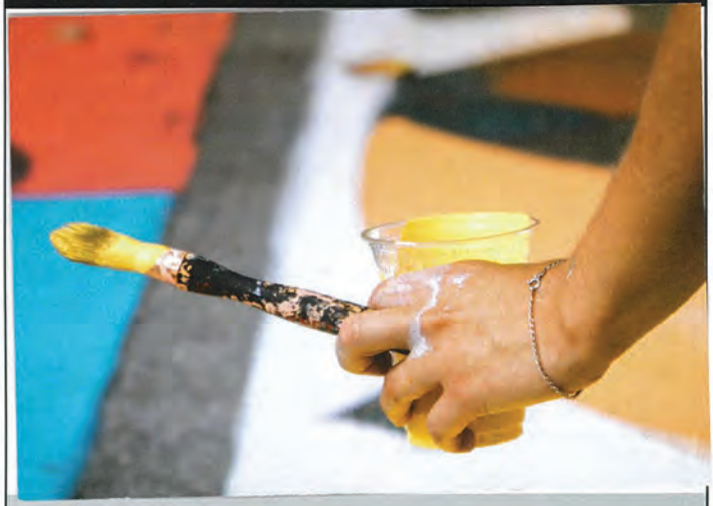

Title: CREATIVITY

This is important because:
Mental stimulation

# Card Sort Activity 1
## What is most important about "home"?

the design clinic

### Picture Card 1

[Place picture card here]

Title:

This is important because:

### Picture Card 2

[Place picture card here]

Title:

This is important because:

### Picture Card 3

[Place picture card here]

Title:

This is important because:

### Picture Card 4

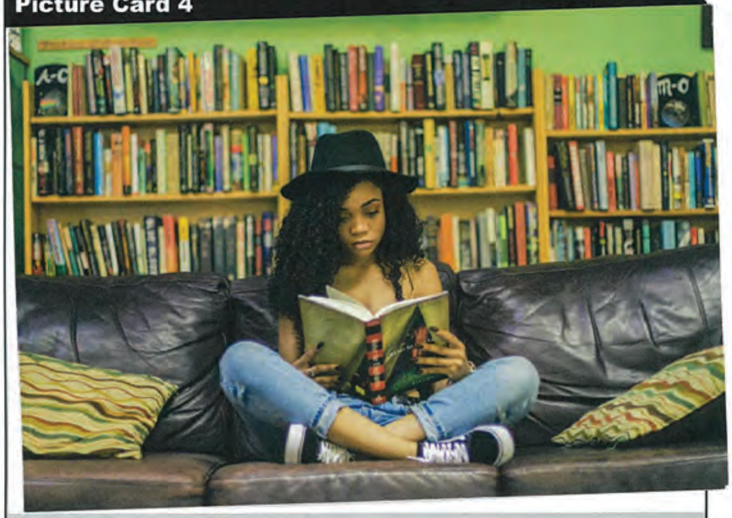

Title: LEARNING & RELAXATION

This is important because: if you stop you're dead

# Card Sort Activity 1

## What is most important about "home"?

the design clinic

### Picture Card 1

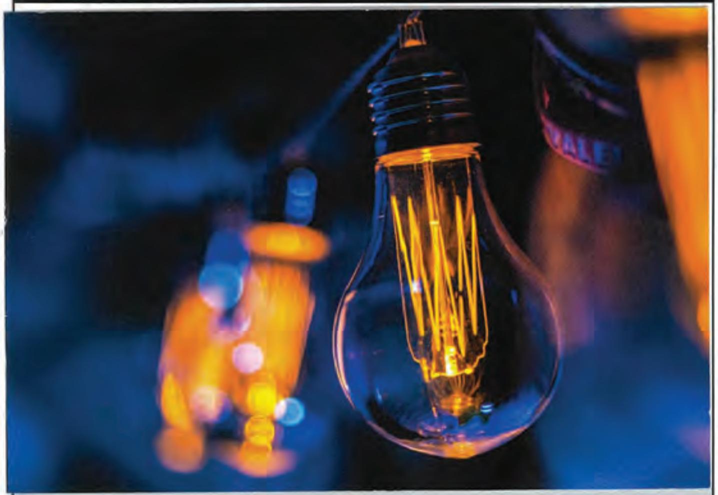

Title: WARMTH & LIGHT

This is important because:

creature comfort
well being
optimism

### Picture Card 2

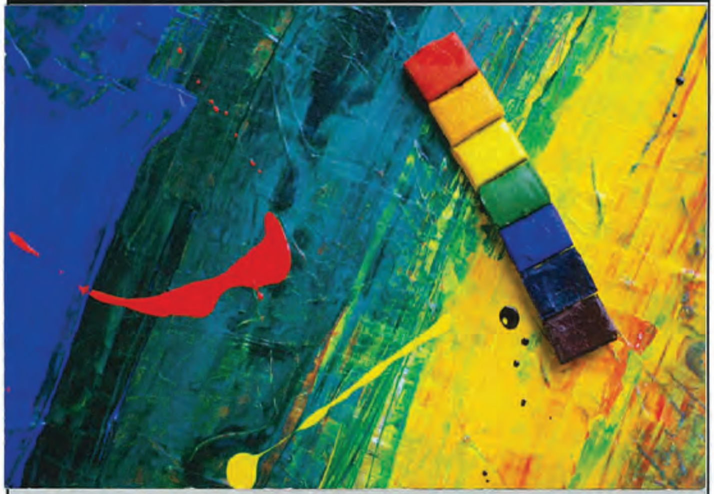

Title: ART & CREATIVITY

This is important because:

spirituality
we are creative beings
good for the soul.

### Picture Card 3

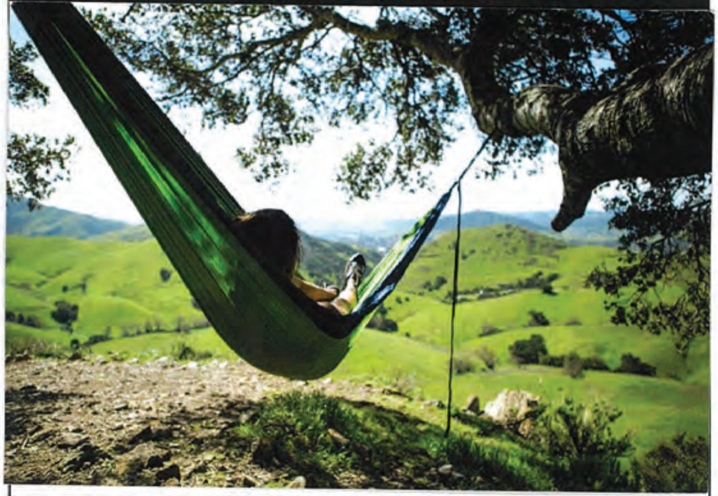

Title: FREEDOM & FRESH AIR + NATURE

This is important because:

it just is!

### Picture Card 4

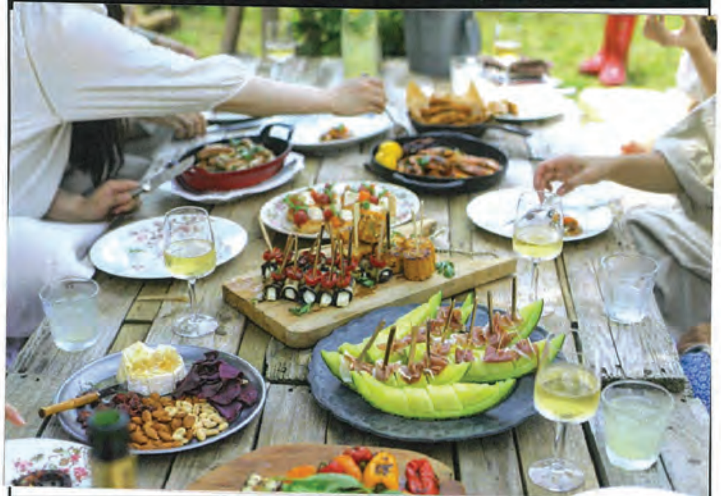

Title: GOOD FOOD & GOOD FRIENDS

This is important because:

Interaction, humour, laughter
culture, conversation, support, ideas

## The most important things about "Home"...

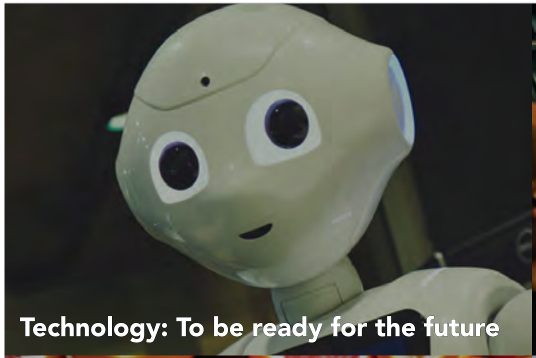
Technology: To be ready for the future

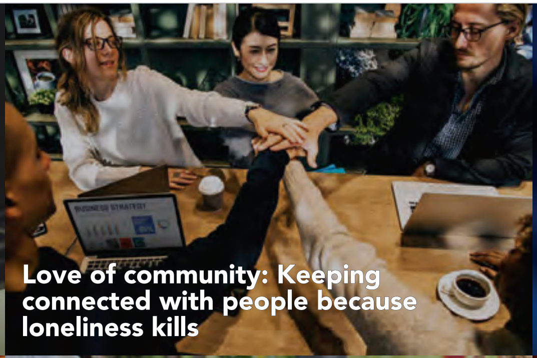
Love of community: Keeping connected with people because loneliness kills

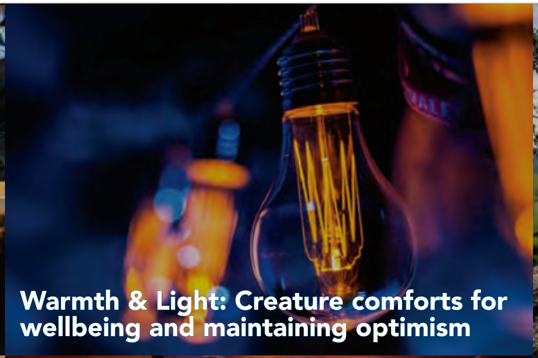
Warmth & Light: Creature comforts for wellbeing and maintaining optimism

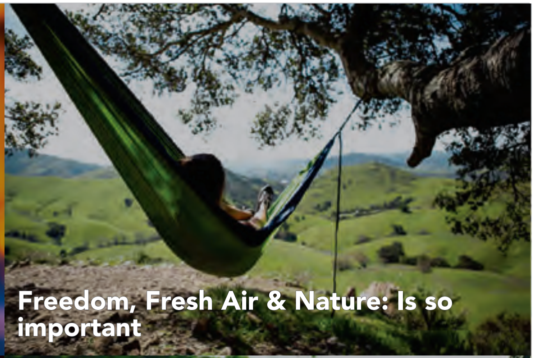
Freedom, Fresh Air & Nature: Is so important

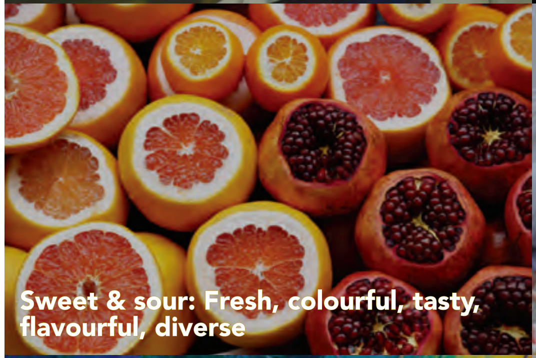
Sweet & sour: Fresh, colourful, tasty, flavourful, diverse

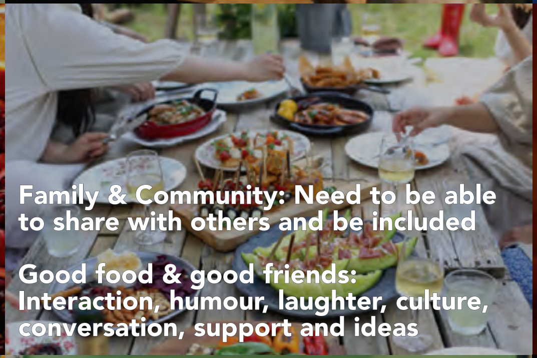
Family & Community: Need to be able to share with others and be included

Good food & good friends: Interaction, humour, laughter, culture, conversation, support and ideas

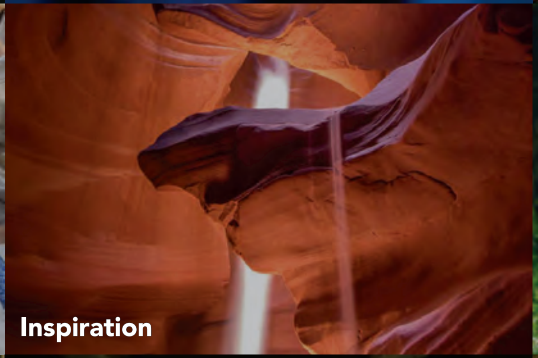
Inspiration

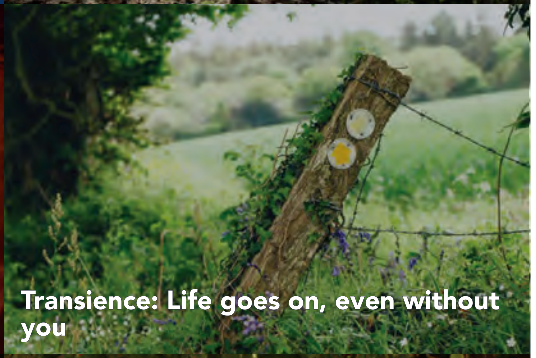
Transience: Life goes on, even without you

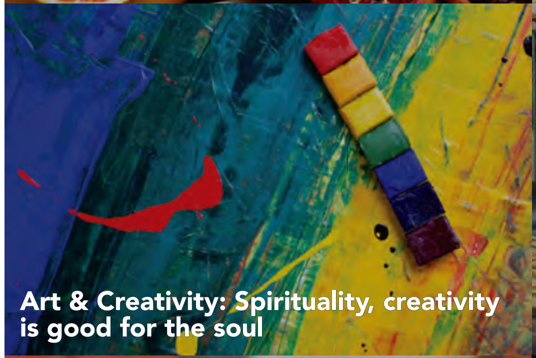
Art & Creativity: Spirituality, creativity is good for the soul

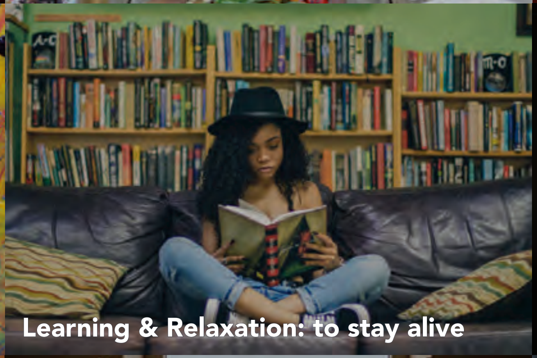
Learning & Relaxation: to stay alive

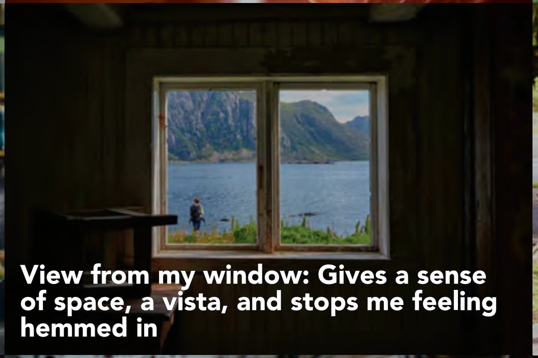
View from my window: Gives a sense of space, a vista, and stops me feeling hemmed in

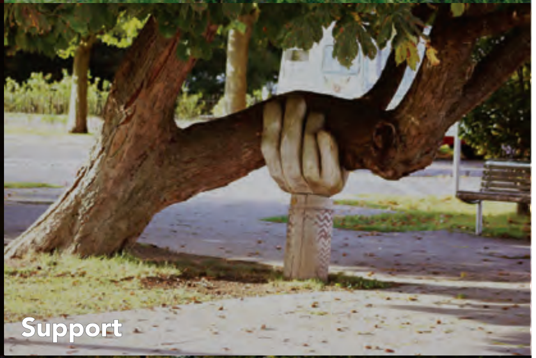
Support

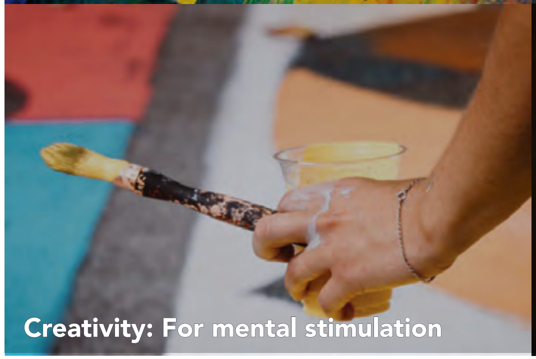
Creativity: For mental stimulation

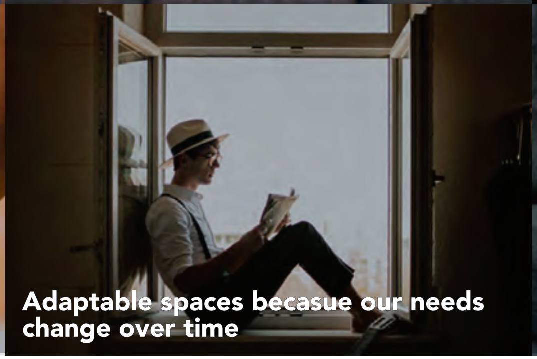
Adaptable spaces becasue our needs change over time

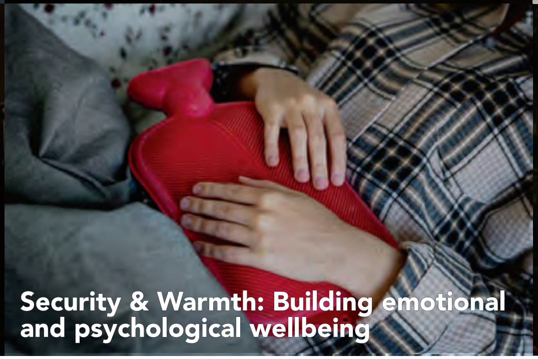
Security & Warmth: Building emotional and psychological wellbeing

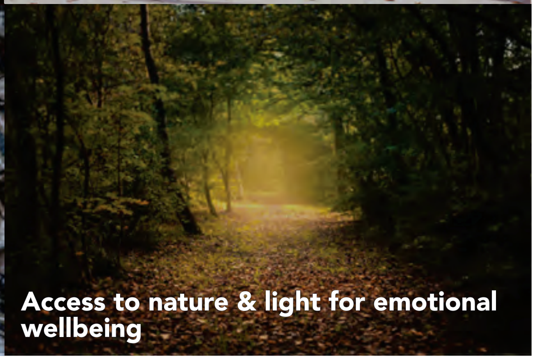
Access to nature & light for emotional wellbeing

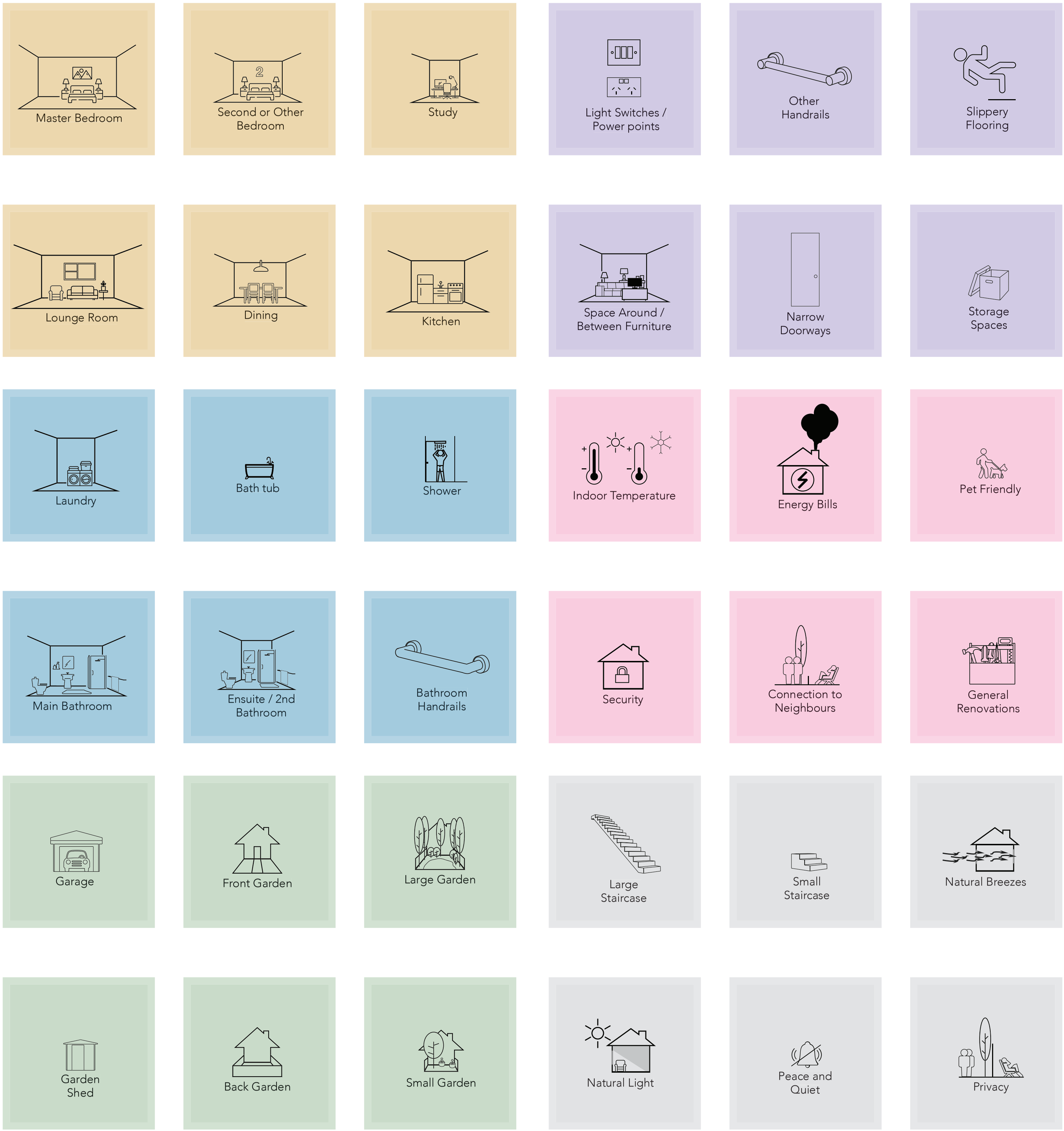
Master Bedroom
Second or Other Bedroom
Study
Light Switches / Power points
Other Handrails
Slippery Flooring
Lounge Room
Dining
Kitchen
Space Around / Between Furniture
Narrow Doorways
Storage Spaces
Laundry
Bath tub
Shower
Indoor Temperature
Energy Bills
Pet Friendly
Main Bathroom
Ensuite / 2nd Bathroom
Bathroom Handrails
Security
Connection to Neighbours
General Renovations
Garage
Front Garden
Large Garden
Large Staircase
Small Staircase
Natural Breezes
Garden Shed
Back Garden
Small Garden
Natural Light
Peace and Quiet
Privacy

# Card Sort Activity 2

## Things I think will need to be adapted as I get older...

**Picture Card 1** — Medium

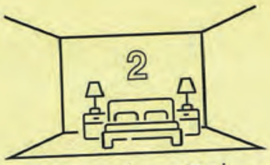

Second or Other Bedroom

When? and Why?
10 years +
Separate Bedrooms

**Picture Card 2** — Easy

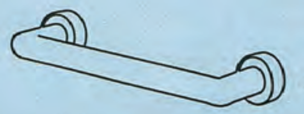

Bathroom Handrails

When? and Why? as soon
Balance deteriates

**Picture Card 3** — Easy

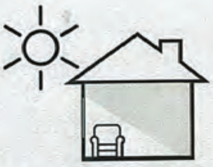

Natural Light

When? and Why?
Now
Sunshine makes you feel better

**Picture Card 4** — Medium

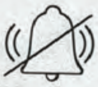

Peace and Quiet

When? and Why?
Now
Keeps me Sane!

**Picture Card 5** — Easy

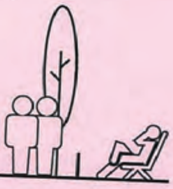

Connection to Neighbours

When? and Why?
Now
Helping, happiness and health

**Picture Card 6** — Hard

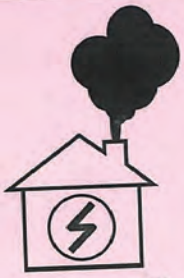

Energy Bills

When? and Why?
Now
Reducing Carbon and Cost

# Card Sort Activity 2

## Things I think will need to be adapted as I get older...

the design clinic

**Picture Card 1**

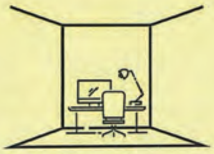

Study

When? and Why? ASAP
SPEND MUCH TIME HERE
NEED TO OPTIMIZE $

Easy

**Picture Card 2**

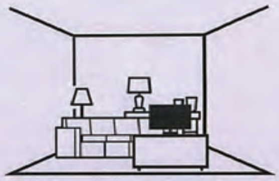

Space Around / Between Furniture

When? and Why? LATER
EASE OF MOVEMENT

Medium

**Picture Card 3**

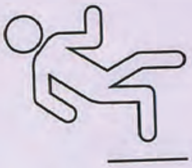

Slippery Flooring

When? and Why? MID-TERM
- BATHROOM

Medium

**Picture Card 4**

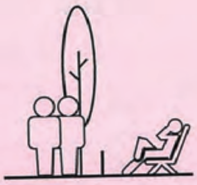

Connection to Neighbours

When? and Why? ASAP.
- I NEED TO TAKE INITIATIVE

Easy

**Picture Card 5**

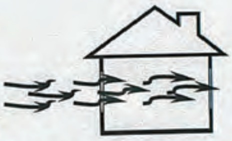

Natural Breezes

When? and Why? SOMETIMES
- HEALTH
- FRESH ODOUR

Easy

**Picture Card 6**

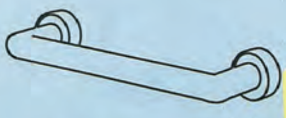

Bathroom Handrails

When? and Why? LATER
- OCCASIONAL BALANCE DEFECTS.

Medium

# Card Sort Activity 2

## Things I think will need to be adapted as I get older...

### Picture Card 1

**Hard**

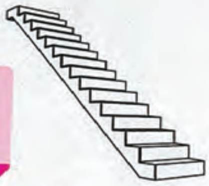

Large Staircase

When? and Why?

Too frail.
To stop me falling.

### Picture Card 2

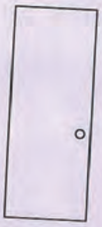

Narrow Doorways

**Hard**

When? and Why?

### Picture Card 3

**Medium**

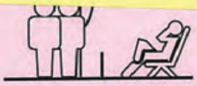

Connection to Neighbours

When? and Why?

Easy, comfortable.
Connections are
~~strait~~ natural.

### Picture Card 4

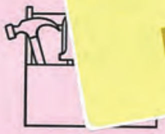

**Medium**

General Renovations
& maintenance

When? and Why?

all the time.
Ongoing. Old house

### Picture Card 5

**Easy**

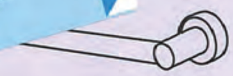

Other Handrails

When? and Why?

? When I become
~~unsta~~ unstable

### Picture Card 6

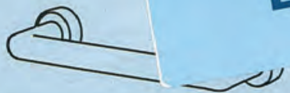

**Easy**

Bathroom Handrails

When? and Why?

?

# Card Sort Activity 2

## Things I think will need to be adapted as I get older...

**Picture Card 1** — **Hard**

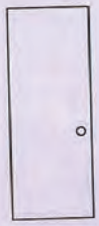

Narrow Doorways

When? and Why?
aspirational – would be difficult and expensive $

**Picture Card 2** — **Hard**

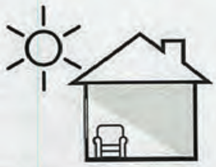

Natural Light

When? and Why?
Medium to long term – don't get much afternoon light in lounge. $

**Picture Card 3** — **Medium**

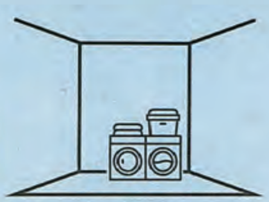

Laundry

When? and Why?
next 2-3 years. Updating!

**Picture Card 4** — **Hard**

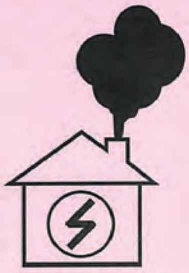

Energy Bills

When? and Why?
Ongoing – [illegible] $
no mains $H_2O$ – all electricity. not solar suitable.

**Picture Card 5** — **Hard**

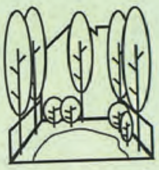

Large Garden

When? and Why?
Current & ongoing Terraced back garden $$

**Picture Card 6** — **Medium**

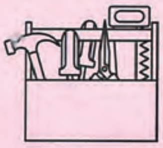

General Renovations
& Maintenance –

When? and Why?
Current – and ongoing maintenance $

# Card Sort Activity 2

## Things I think will need to be adapted as I get older...

the design clinic

**Picture Card 1**

**Easy**

Bathroom Handrails

When? and Why?

For safety & stability.

**Pict[illegible]**

**Easy**

Other Handrails

When? and Why?

To assist with mobility, safety etc.

**Medium**

**Hard**

Shower

When? and Why?

When I am no longer able to shower myself. will need to change the shower head to hand held. Also, may need to increase size of shower frame.

**P[illegible]**

**Hard**

Narrow Doorways

When? and Why?

All doors are standard size. They do not easily accomodate walking frames or wheelchair access. Door frames would need to be wider.

**Picture Card 5**

**Hard**

Peace and Quiet

When? and Why?

Peace & quiet relates to neighbours. Current neighbours on both sides are noisy! Not easily addressed.

**Picture Card 6**

**Easy**

2

Second or Other Bedroom

When? and Why?

Less furniture in the room for access & safety

# Card Sort Activity 2

## Things I think will need to be adapted as I get older...

the design clinic

**Easy**

**Picture Card 1**

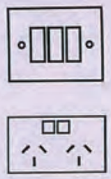

Light Switches / Power points

When? and Why? Some Power Points low + behind furniture. when getting frail

EASY.

**Picture Card 2**

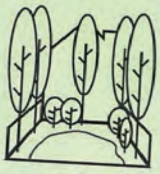

Large Garden

When? and Why?
Garden hard to maintain when older. Needs to be low maintenance.
MEDIUM

**Picture Card 3**

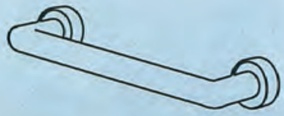

Bathroom Handrails

When? and Why?
Expect to need these as older.
EASY

**Picture Card 4**

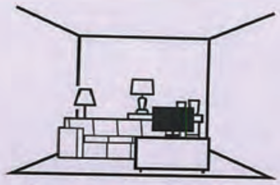

Space Around / Between Furniture

When? and Why?
Some tables in middle of floor → dangerous to tripping over.
EASY

**Picture Card 5**

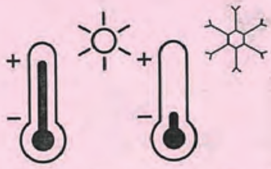

Indoor Temperature

When? and Why?
Currently only half house heated. Need full house heating. EASY [illegible]

**Picture Card 6**

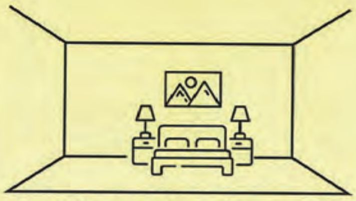

Master Bedroom

When? and Why?
Current bed mattress is very heavy. will be harder to move around when older.
EASY.

# Card Sort Activity 2

## Things I think will need to be adapted as I get older...

### Picture Card 1 — Hard

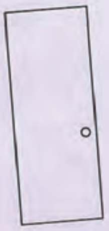

Narrow Doorways

When? and Why?
Might be necessary when wheelchair is used
When? hopefully never

### Picture Card 2 — Medium

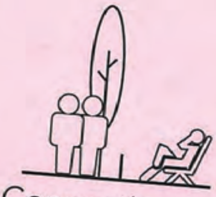

Connection to Neighbours

When? and Why?
Asap. Next door property is not occupied regularly
No connection to other side
Need to establish contact.

### Picture Card 3 — Medium / Hard

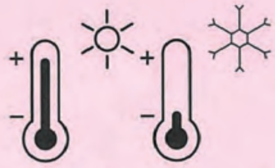

Indoor Temperature

When? and Why?
Asap. ~~the~~ Too cold in winter

### Picture Card 4 — Hard

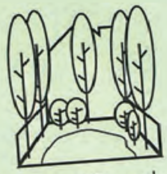

Large Garden

When? and Why?
When I can't work in it anymore. ~5 years to reduce
Too much work / steep driveway

### Picture Card 5 — Easy

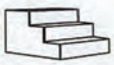

Small Staircase

When? and Why?
When necessary.
Needs hand rail.

### Picture Card 6 — Easy

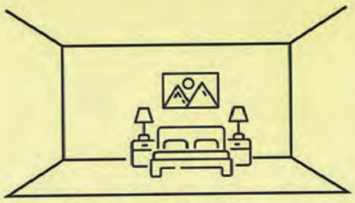

Master Bedroom

When? and Why?
Asap. Needs a wardrobe

# Card Sort Activity 2

## Things I think will need to be adapted as I get older...

the design clinic

### Picture Card 1

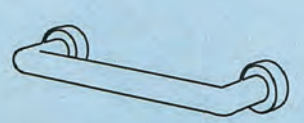

Bathroom Handrails

Medium

When? and Why?
?? In next 10 years.
To avoid slipping hazard & falls
COST $

### Picture Card 2

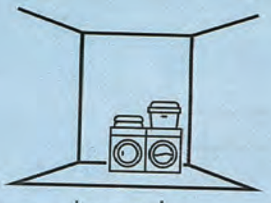

Laundry

Hard

When? and Why?
Soon !!
Laundry has no floordrain.
Access to clothesline is not direct. steps.
COST $

### Picture Card 3

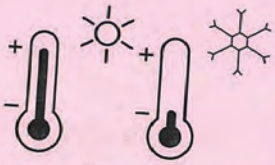

Indoor Temperature

Medium

When? and Why?
Sooner.
Timber house with raised floors & insufficient insulation. INSULATION floors ceiling.
COST $

### Picture Card 4

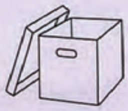

Storage Spaces

Medium

When? and Why?
Insufficient & inaccessible storage. Medium $ COST.

### Picture Card 5

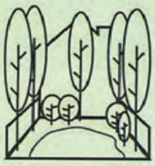

Large Garden

Hard

When? and Why?
Over next 10 years.
Hard because lots of work. and cost $.
→ low maintenance.

### Picture Card 6

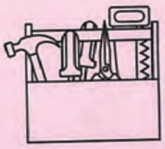

General Renovations

Medium

When? and Why?
Steps at back & front verandahs.
Locks & windows.
COST $
Water tanks.

# Card Sort Activity 2

## Things I think will need to be adapted as I get older...

the design clinic

**Hard**

**Picture Card 1**

Front Garden

When? and Why?
- soon
- low maintenance
- out door dining etc.
- finance

**Picture Card 2**

Storage Spaces

When? and Why?
- soon
- uncluttering
- tidyness

**Medium**

**Medium**
[illegible]

**Picture Card 3**

Garden Shed

When? and Why?
- soon
- unclutter
- safety

**Picture Card 4**

Security

When? and Why?
- in a few years
- safety
- $?

$ **Medium**

**Easy**
buy & install

**Picture Card 5**

Bathroom Handrails

When? and Why?
- in future
- safety in shower

**Picture Card 6**

Energy Bills

When? and Why?
- now
- [illegible] for decreasing global warming

**Easy**

# Card Sort Activity 2

## Things I think will need to be adapted as I get older...

**Picture Card 1** — Medium

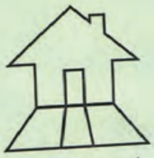

Front Garden

When? and Why? Soon currently a carpark to be Nth facing garden

**Picture Card 2** — Medium

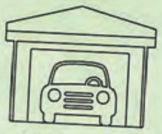

Garage

When? and Why? soon → Man Cave

**Picture Card 3** — Hard

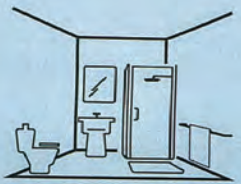

Ensuite / 2nd Bathroom

When? and Why? need a 2nd toilet

**Picture Card 4** — Hard

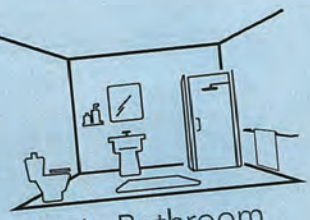

Main Bathroom

When? and Why? reno. to include a bath

**Picture Card 5** — Easy

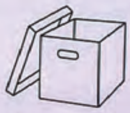

Storage Spaces

When? and Why? more & suitable storage kitchen & outside

**Picture Card 6** — Medium

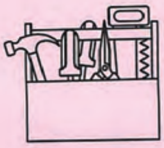

General Renovations

When? and Why? ongoing to create 2nd living/music space

# Card Sort Activity 2

## Things I think will need to be adapted as I get older...

the design clinic

**Picture Card 1**

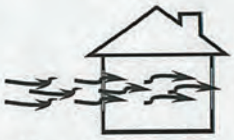

Natural Breezes

When? and Why?
Hard to do as we live in a sheltered area

**Picture Card 2**

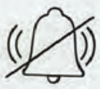

Peace and Quiet

When? and Why?
Metalica

**Hard**

**Pi[illegible]**

[Place card here]

When? and Why?

**Easy**

**Pictu[illegible]**

[Place card here]

When? and Why?

**Picture Card 5**

[Place card here]

When? and Why?

**Picture Card 6**

[Place card here]

When? and Why?

# Card Sort Activity 2

## Things I think will need to be adapt[illegible] as I get older...

**Hard** — $ $ $

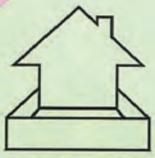

### Back Garden

When? and Why?
Retaining walls, slopes, steps - soon

**Hard** — Don't want to change

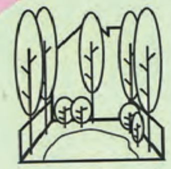

### Large Garden

When? and Why?
Too big to look after. but don't want to lose

**Easy**

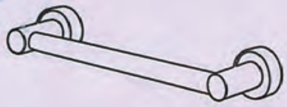

### Other Handrails

When? and Why?
Along long hallway, 2nd toilet - down the track

**Easy** ←

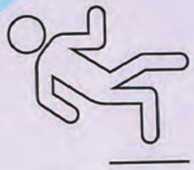

### Slippery Flooring

When? and Why?
Down the track - as mobility decreases - hand rails to assist

**Hard** — $ $ $

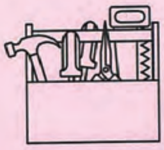

### General Renovations

When? and Why?
Neither of us able to do as we age - painting replace ceiling etc - when can afford.

**Medium**

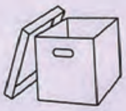

### Storage Spaces

When? and Why?
Reduce clutter - when mum has died so we can lose her stuff.
