# Supplementary material for: Self-assessment of the home environment to plan for successful ageing: Report from a digital health co-design workshop
Source: PLOS Digit Health. 2022 Jul 7;1(7):e0000069. doi: 10.1371/journal.pdig.0000069 (PMC9931232; doi:10.1371/journal.pdig.0000069)
Supplement: S2 Appendix — (PDF) [file pdig.0000069.s003.pdf]

Things I think will need to be adapted as I get older...

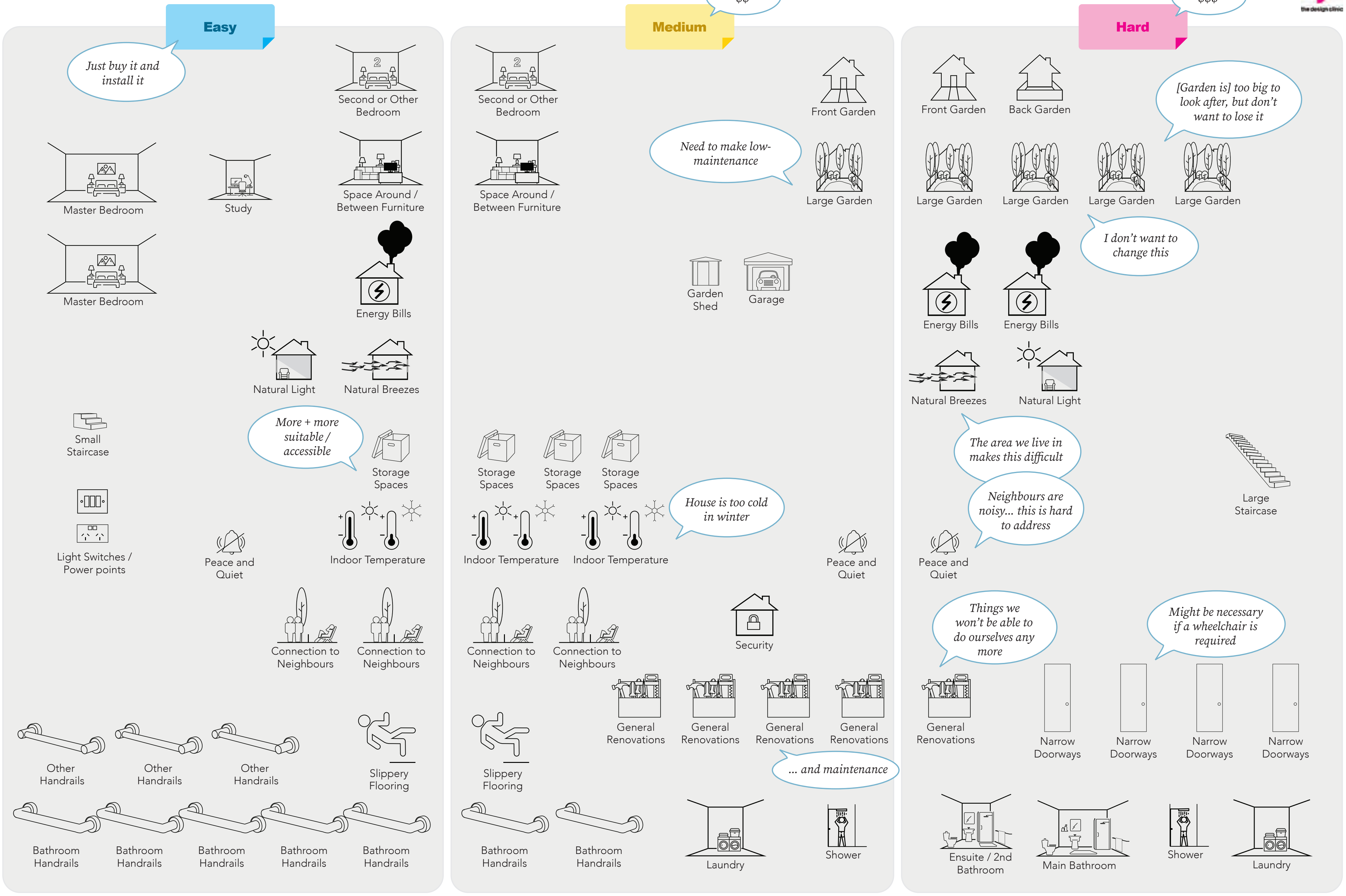

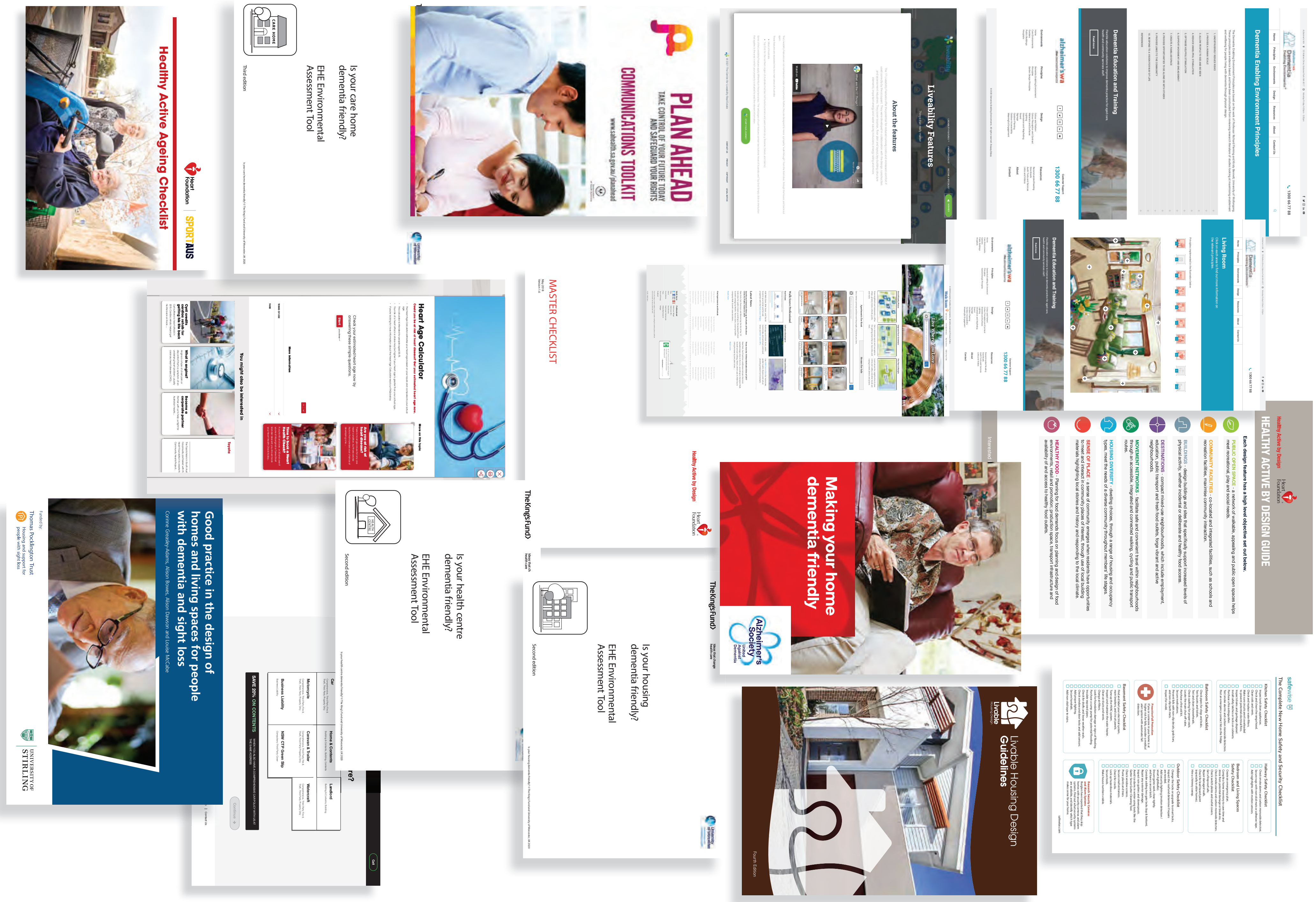
Healthy Active Ageing Checklist
Heart Foundation
SPORT AUS
Is your care home dementia friendly?
EHE Environmental Assessment Tool
Third edition
PLAN AHEAD
TAKE CONTROL OF YOUR FUTURE TODAY AND SAFEGUARD YOUR RIGHTS
COMMUNICATIONS TOOLKIT
www.sahealth.sa.gov.au/planahead
Liveability Features
About the features
Dementia Enabling Environment Principles
Dementia Education and Training
1300 66 77 88
Living Room
Heart Age Calculator
You might also be interested in
MASTER CHECKLIST
Healthy Active by Design
HEALTHY ACTIVE BY DESIGN GUIDE
Making your home dementia friendly
Alzheimer's Society
Good practice in the design of homes and living spaces for people with dementia and sight loss
Corinne Greasley-Adams, Alison Bowes, Alison Dawson and Louise McCabe
Thomas Pocklington Trust
UNIVERSITY OF STIRLING
Is your health centre dementia friendly?
EHE Environmental Assessment Tool
Second edition
The King's Fund
Is your housing dementia friendly?
EHE Environmental Assessment Tool
Second edition
Livable Housing Design Guidelines
Fourth Edition
safewise
The Complete New Home Safety and Security Checklist

Self-assessments review

What works and what doesn't?

Things that work:

Vou site easy to use

LEGEND IS GOOD TO CATEGORIZE INFO

Coding with ticks is good & useful

Would help to include... GRAPHICAL INFO + DASHBOARDS

Heart foundation website Health easy to use + understand Didn't agree with answer

CHECKLISTS ARE USEFUL

Websites that engage

---

text far too small

images also far too small

TOTAL DISENGAGEMENT FROM FIRST IMPRESSION

Publications that ~~too~~ look home/office made

Massive Fail!

- Font size excessively small (c. 6.)
- Impossible to read

White text on a coloured background is hard to read

CHECKLIST:

- Very expensive options
- Very fearful tone
- Some useful suggestions
- Slightly unclear if existing/new purchase/new home

- Academic
- Academic language
- Font too small & too faint
- assume it's not for consumers??
- Is it an abstract?

JARGON !!

[illegible] ur group but not for others, record it in both categories

Self-assessments review
What works and what doesn't?

Things that work:

*Images & pictures
White space (text) —
icons — encourage me to read.

* New ideas to try.
* Videos to show ideas & stories
* Clear contents info & easy to follow links or directions so you don't get frustrated going around in circles & reading irrelevant info.

IDEAS

* A regular magazine with tips & ideas — and opportunities to contribute — real people
* (Newsletter in inbox — email or hardcopy options so it works for different people.
* Use images
* Ask questions so people can put their own ideas & experience in — this also mitigates against "talking down".

Things that don't work:

* Too many words
* Font/text too small
* Lack of contrast/colour
* Too much text — not broken up
* If it looks like too much work, I don't want to read it
* Talking down to older people — patronising tone
* Links that take you around in circles & ultimately provide no information e.g. most govt. websites

Note: If something works for some in your group but not for others, record it in both categories

(TEXT)
"Making your Dementia ~~Home~~ Friendly".
Useful info.
Images & icons encourage reading.

One way to avoid "talking down" is to include questions as well as giving infomation – so readers think for themselves & actively participate

(APP)
App. Limited relevance.

Fairly easy to navigate

Full of "known knowns"

(TEXT)
Icons are helpful. ....
Handy to have a smart device.
(Content is useful.

(TEXT)
Referring to other publications is distracting
e.g. refer to factsheet 437.
(But can also be useful.)

(TEXT)
Use different font & sections & white styles to break space to define up & different levels of types of info.

Images / pictures encourage to read. (TEXT)

(TEXT)
Text too small – too much text – needs to be broken up.
~~use diff~~

Self-assessments review

What works and what doesn't?

Things that work:

It was not a chore to use

Heart Age App. Clear
Easy to use
~~Access to explanatory info could be better - put in centre not side~~
Seemed a bit simple for accurate results

Good font size (12/14)
Like use of gold/silver standard
Quite technical - OK for people that can read plans etc. Need to read with building designer

Like webpage rather than App

Things that don't work:

Should have indicative cost time frame

Easy to navigate good info
~~indicative cost~~ ??
Aimed more at carers than elderly

lot of superfulous info on side
~~Clear~~
Put explanatory info in centre not to the side
seemed simple for accurate results

Font colour
Need to go off page for any info
Need to download before beging

Note: If something works for some in your group but not for others, record it in both categories

T. O. C
Looks good -
until you get
into it

Trustworthy??
trust me I am the
Govt!!
Guide has better
layout because
it is clear
sumerised
designed

Misleading
title
- NOT A CHECKLIST
- guidelines

Someone
elses assumptions
about what matters
to me

Based on
assumptions

Not for people
for services

What does
300th Most Walkable
neibourhood mean?

Font size - too small

limited coverage
for each room/
garden

Evidence
- from where?

Not
Able to select
more than 1
option
- Youii site

Single platform
(layout)
Font - too small
Wordy / Busy
too much info
in a small space

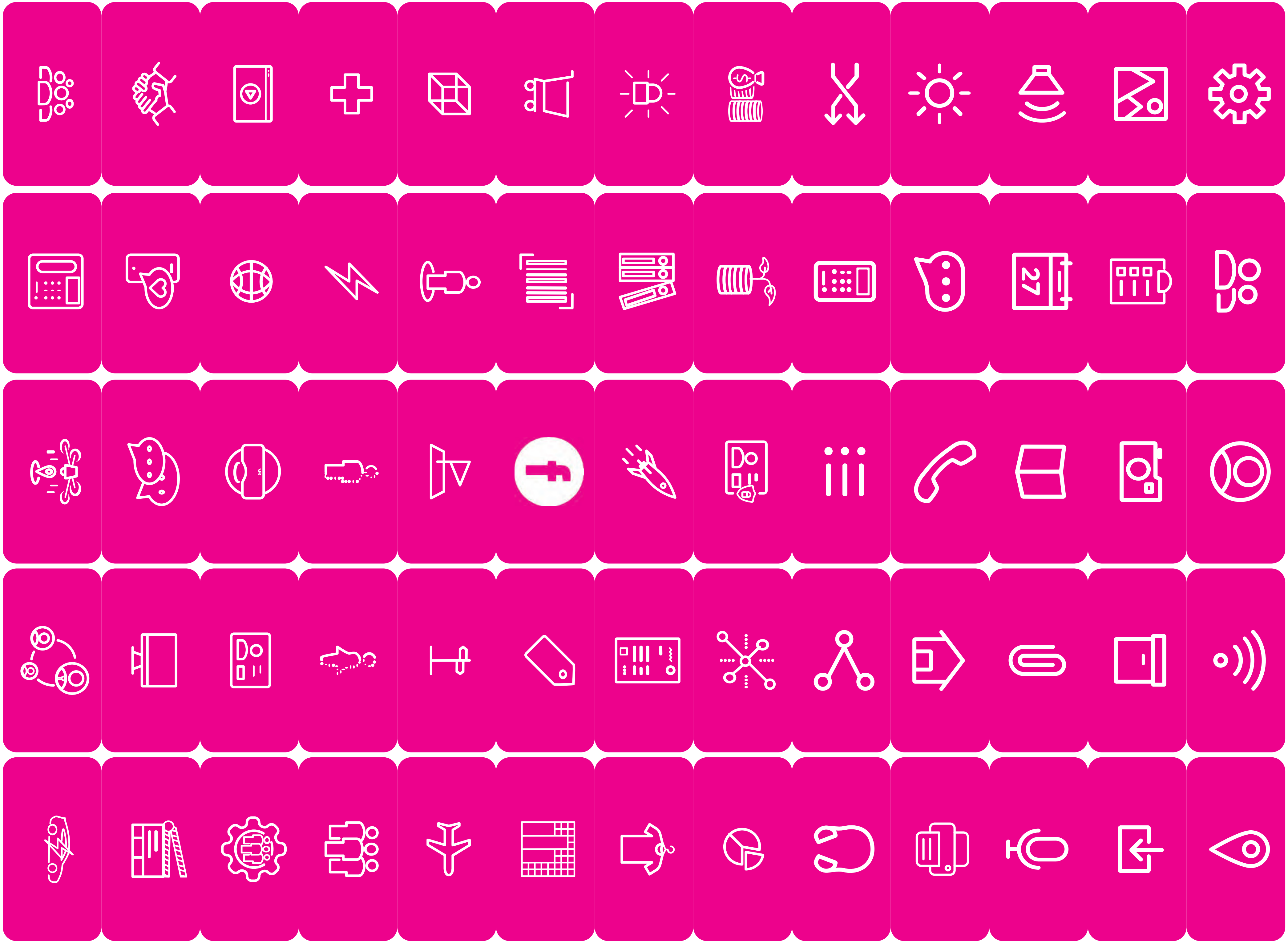

Features Brainstorm

Features in a self-assessment tool...

the design clinic

(1) Profile

(1) Profile

(2) location environment Community

(3) House style/ layout

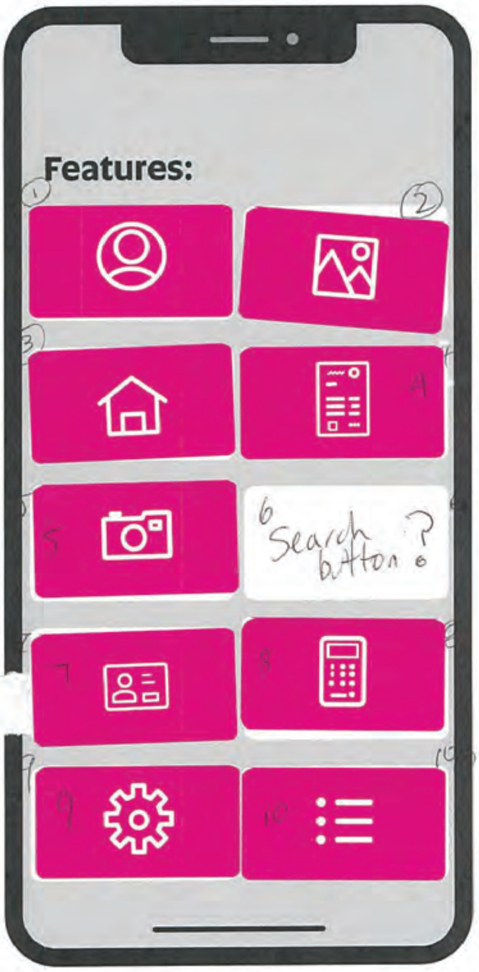

(4) Design ideas/ guidelines checklist

(5) Image set

(6) Search / Help

(7) Useful contacts

(8) Costings inc. enviro. cost.

(9) Settings

(10) – Menu

Would also like consideration of environmental costs

→ ability to reuse & reduce resource use etc.

Features Brainstorm

Features in a self-assessment tool...

Home page
- back to the start when you are lost or confused

Live chat
- preferably with a real person.

Download
- useful info to keep

SETTINGS
- to customize font size, colors, muzak

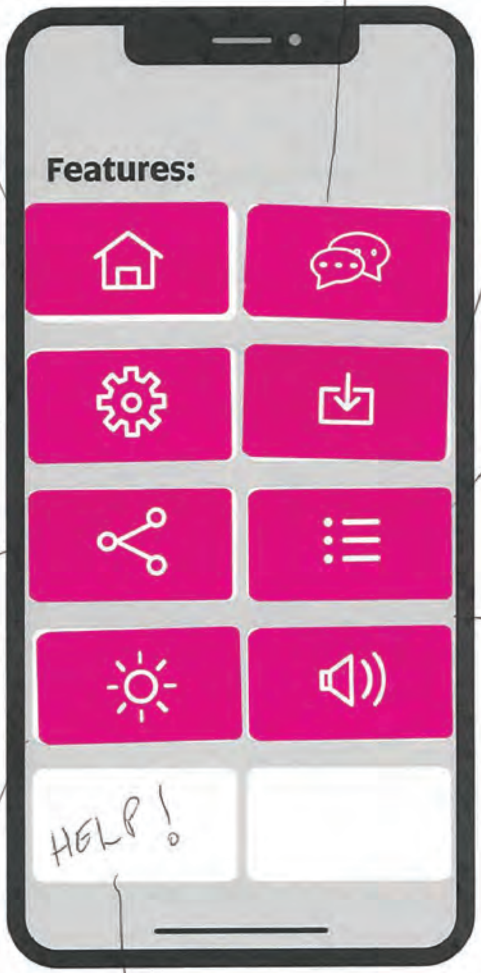

MENU
- select room / equipment etc. submenu - what do you want to do
  - look
  - select
  - cost

Share
- ability to share info with others

Speaker - listen not read (safety as walking around house)

Brightness
- dependant on time of day / personal needs

Help function
- access to help
- written / spoken

Features Brainstorm

Features in a self-assessment tool...

HELP (MORE INFO)
- Links to other pages that provide more detailed information.

~~A~~ NO IN-BUILT "SPONSOR" ADS THAT SLOW THE APP DOWN AND MAKE IT FRUSTRATING TO USE

VOLUME CONTROL (when Audio provided)

ABILITY TO DOWNLOAD INFORMATION FOR LATER REFERENCE

ABILITY TO SELECT DOWNLOADED MENU. (AND SUB MENUS.)

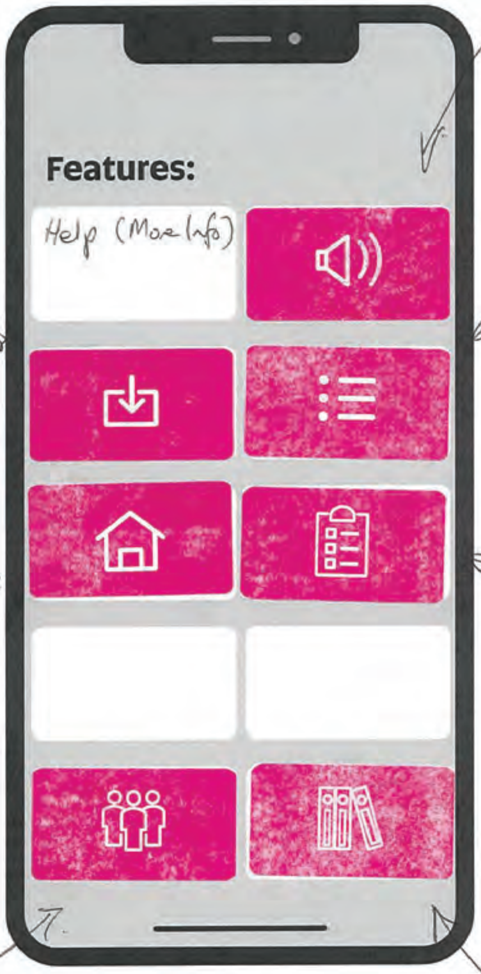

ABILITY TO RETURN TO THE HOME PAGE (when needing to resume)

TEXT THAT IS CONCISE, CLEAR, + WELL SUMMARISED. NOT WORDY.

ABILITY TO HAVE SHARED PROFILES e.g. so that can be shared between family members.

ABILITY TO LINK TO (OR DOWNLOAD) OTHER DETAILED REFERENCES e.g. LINKS TO DIY VIDEOS.

Features Brainstorm

Features in a self-assessment tool...

the design clinic

My Profile.
- Personal details.
- Link to Health data.
- Family Links.

Notes / Ideas.
- Things I have thought of that would be useful.
- Things I am having troubles using/doing.
- What would make life easier if it was changed /improved.

Health.
→ Links to
- Medical information.
- Doctors
- Medications.
-

Travel / Holidays.
- Bucket list.
- Fav. Hotels
- Fav. Places
- Planning.

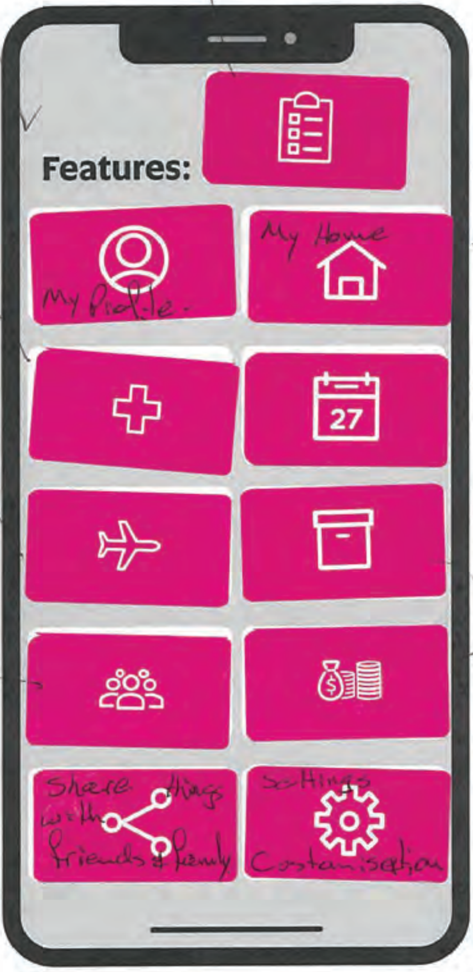

My Home.
- Link to all sub feature areas
  → Livability
  → Technology
  → Safety.
  (Links to relevant websites.)

Calendar.
- Appointments.
- Planning.

Social
Links to Social Media
Contact lists.
Email.
Photos.

Finances.
- Banking.
- Investments.
- Social services.

Storage.
- Photos
- Documents
- ideas.

Latest News on Aging.

Generally a mix of:
- My information
- My Current situation.
- Things to make life easier
- Asparational things → travel.
-

Cross reference all input to give direction and/or ideas going forward.
→ What is available
→ ideas/alternatives.

Features Brainstorm

Features in a self-assessment tool...

This is more general — not just about self-assessment, but something to use at home every day

TAILORED EXPERIENCE — e.g. "BALANCE" (meditation app.)

QUESTIONS — "Choose your own adventure" style of app. to make it really relevant to each person now & in future.
→ Input own IDEAS
Make the app a continuous co-design project — so it keeps evolving.

STORAGE SPACE

LINKS & BOOKMARKS on info & articles to read later &/or keep for reference e.g. recipes, tips, instructions, info.

+ ABILITY to print screens.

GARDEN & GARDENING support & info.
Also → a way to record ideas on the go.. e.g. voice memo.
e.g. for gardening & fishing.

NAVIGATION
Having a clear menu with links to easily navigate to information I want (& back).
'AGE - FRIENDLY NAVIGATION'

Space for personalised diary to input ideas, inspiration, random thoughts, spiritual diary.

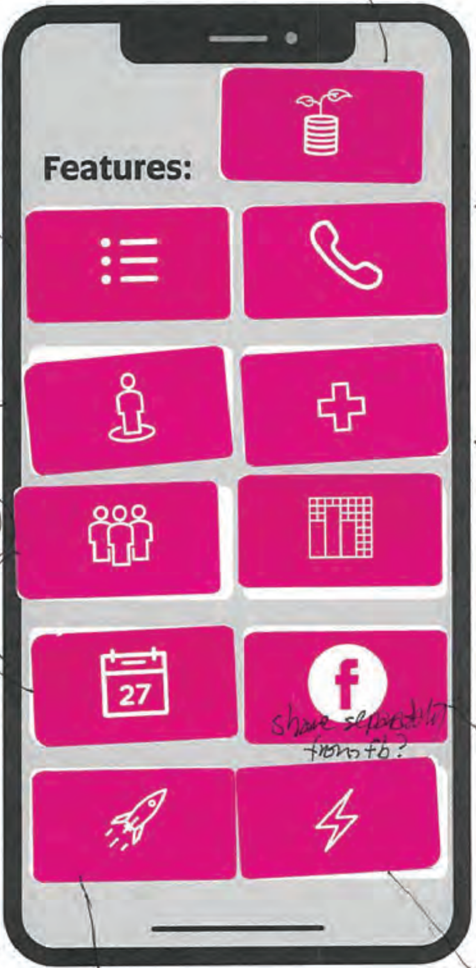

Being able to phone friends, urgent/emergency numbers, get technical help, talk to a real person!
Who do I need to contact? Important numbers. Friends & family. How can I access easily? (instant dial, look up, ...?)

$ BANKING & FINANCE

MEDICAL / HEALTH APP.
Info on medications when to take / renew prescriptions. Make medical appointments incorp. MEDICALERT.
LINKS to evidence based research
Also for PETS (vet).
Share with friends & family on facebook

CALENDAR for everyday reminders of appointments, things to do etc. (+ maps)
LINK to other features of the app.
NOTIFICATIONS — by sound & pop-up messages (e.g. birthdays, appointments)
copies of tickets / passports.
SHOPPING LIST

share separately from fb?

FAMILY & FRIENDS
— address book with direct dial links
personalise with photos, quotes etc.
integrate with address book / contacts.

COMMUNICATION NEEDS

TRANSPORT OPTIONS
Timetables, car servicing, taxi companies, reminders for bookings (link to calendar)
How do I get around? How might this change? What do I need to know?

ELECTRICAL appliances with codes, warranties, manuals, contact info for repairs, energy usage, & bills. (what will it cost?)
What do I need to keep up to date with re electrical appliances & house maintenance?

Features Brainstorm

Features in a self-assessment tool...

Infos I need about Gardening
Reminders when to plant what
tailored to my garden

~~[illegible]~~ Friends
Communication

My home
improvements

New projects

or

My music
+ music suggestions

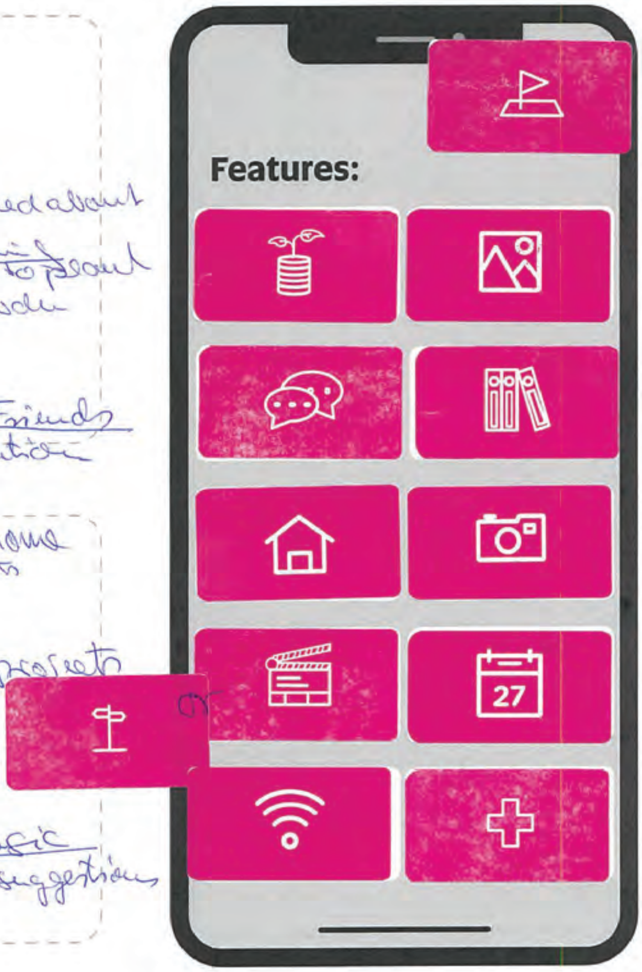

Health – fitness, relaxation
meditation

Travel Schedules

Studying My Studies
Study deadlines, Reminders
Study related webpages

Artistic, Images, my
artwork, Art

My appointments

Doctors
medical support
life support
Emergency Support

Features Brainstorm

Features in a self-assessment tool...

the design clinic

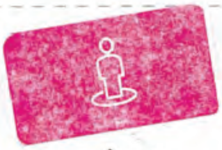

My App
that would contain all
of the below protected
by face recognition

Travel diary/Itenary
Calendar Passports
Travel Card

Plants maintenance

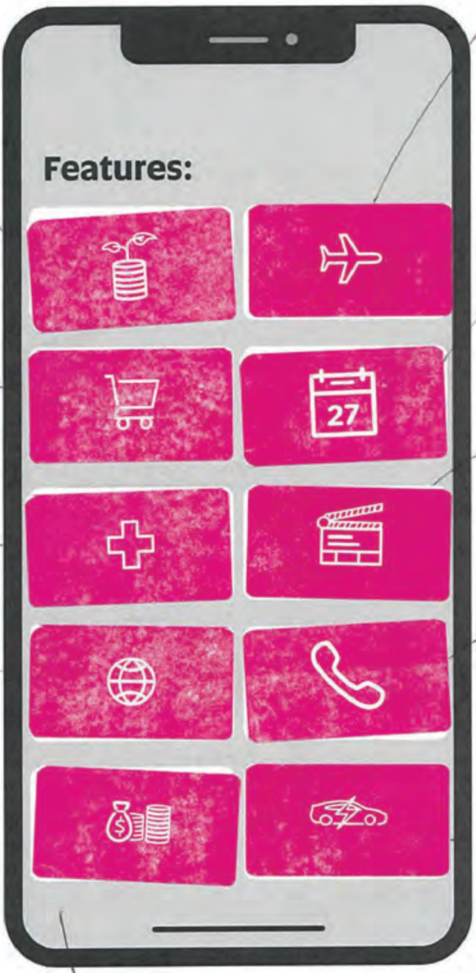

Shopping lists
and connection to Supermarkets

Calendar - dates
that automatically update to
partner or friend

Filing of reports
Dividend reports

Ambulance + Medical

reminding to call
friends

Access to www easily
and a reasonalby priced.

Car Service calendar

Banking - payment
of bills

Features Brainstorm

Features in a self-assessment tool...

APP MUST
1. IDENTIFY WHAT TO CONSIDER.
2. LINK TO RESOURCES (CONTACTS, LINKS, DOCS)
3. STORE WORK TO DATE.

CHECKLIST FOR ITEMS OF PARTICULAR INTEREST.

CONTACTS

ATTACH NOTES IMAGES DOCS.

LIGHTING

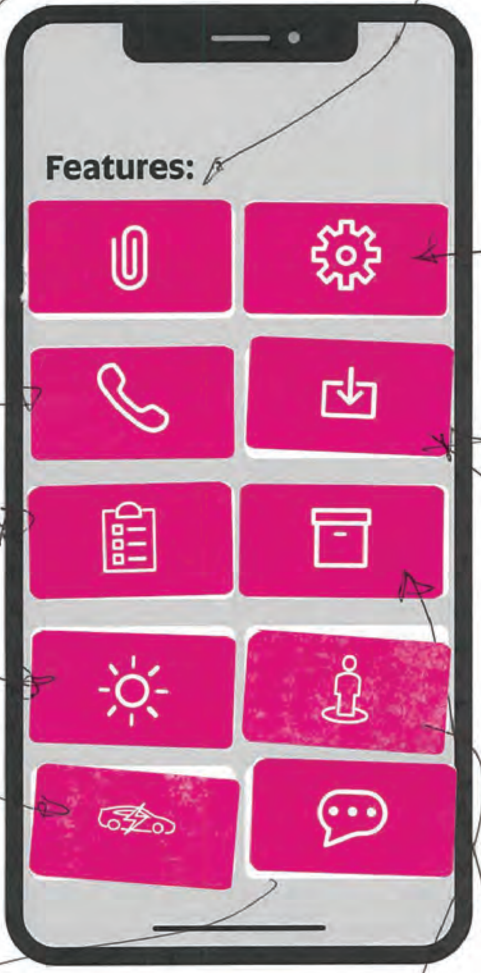

SETTING
- SELECT ICONS
- FONT SIZE COLOUR BACKGROUND

TRANSPORT.

DOWNLOAD relevant docs

OTHER OPTIONS

WALKABILITY

STORAGE

Features Brainstorm

Features in a self-assessment tool...

the design clinic

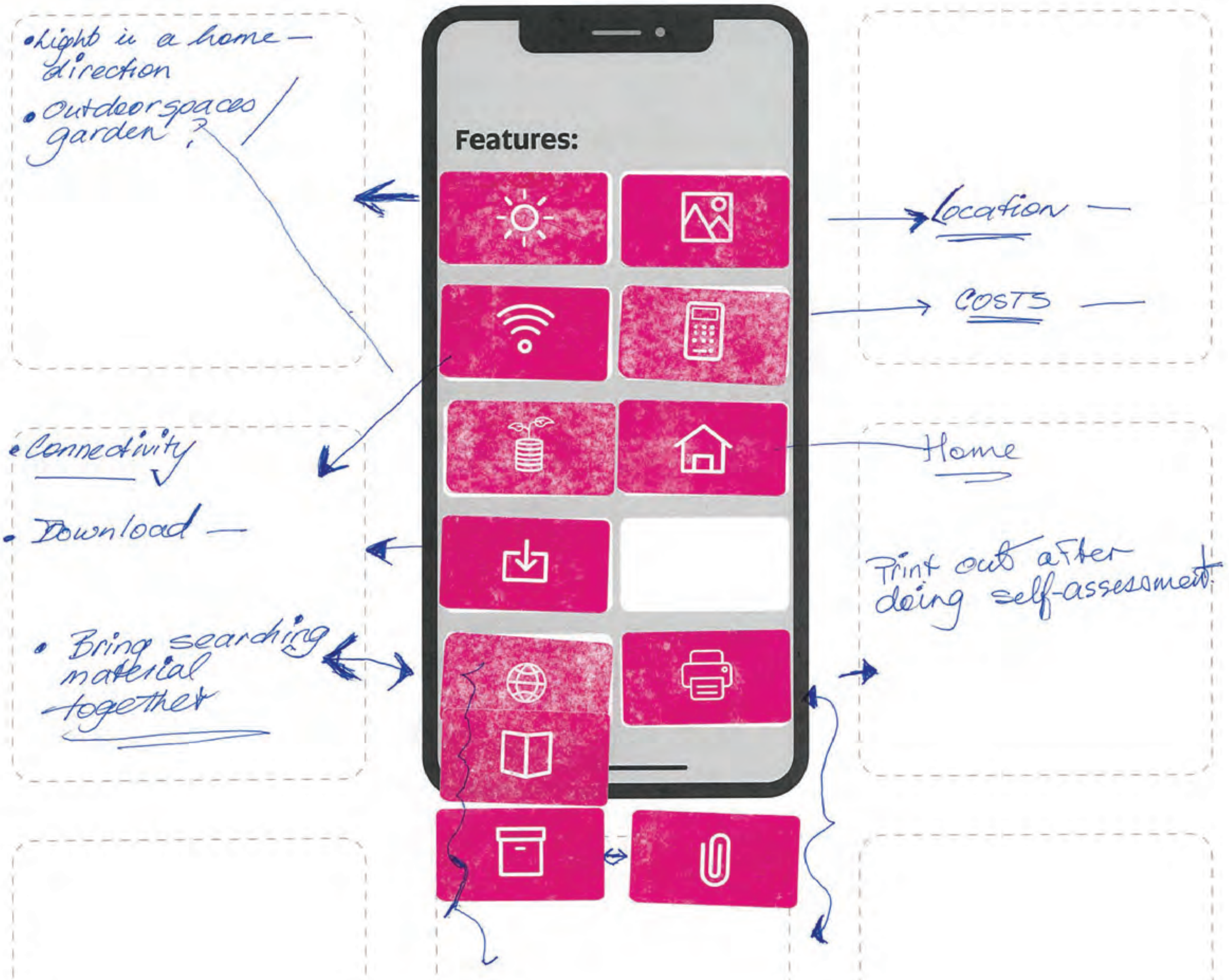

Features Brainstorm

Features in a self-assessment tool...

Hover over button gives brief description - make the icon not platform specific

1: Home - go back to

6: Personalise Profile

7 Download / store Info

2 Pick your topic
How to
Indicative cost
Difficulty level

Importance — maybe 3 should be above 2

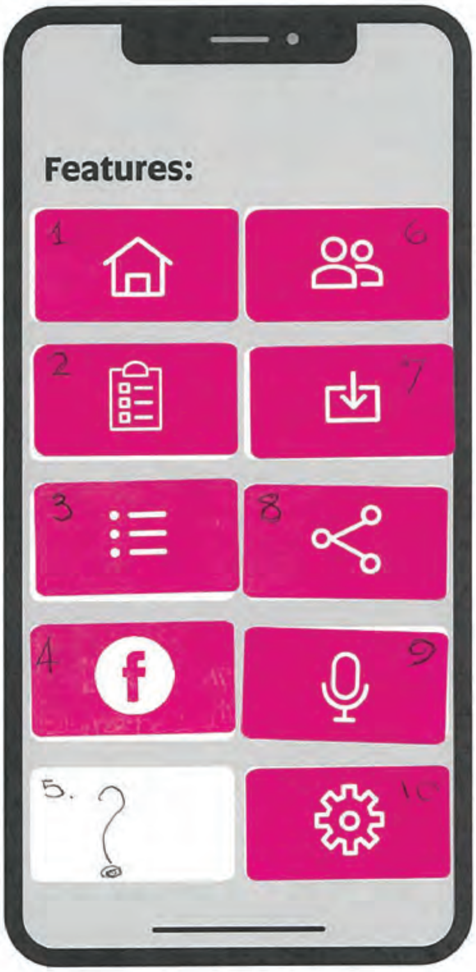

8 Share information
Send to email / text

3 Pick your location
Room
Garden
Shed

9 Audio - reads the page
Text to Speech / Translate

4 Stab yourself repeatedly through your eyeball into your brain

5 Help

10 Toolbox / Settings
Size Colour
Font
Language
Sound Volume
Date / Time

Features Brainstorm

Features in a self-assessment tool...

the design clinic

Features:

User profile	Save what I've done to continue later.
Each step/question to have option to investigate further.	Where do I compare ~~fit~~ with others?
Automatic login with username.	~~Reports~~ ~~Storage~~ Guide to researching
Suggestions for how to fix any problems	~~Share~~ Share results with others.
Choice of language	Print

Features Brainstorm

Features in a self-assessment tool... + its suitability for ageing

1. Checklist of features.
Criteria against which I am assessing my home's suitability/optimisation for my ageing well.
eg. room by room?

~~4. Searchable Database~~
People using AT.

6. ~~other~~ Sharing.

2. Latest Research/Evidence on home design, technology aided homes, safety + security etc. (Expert input – who does not have financial benefit from advertising)

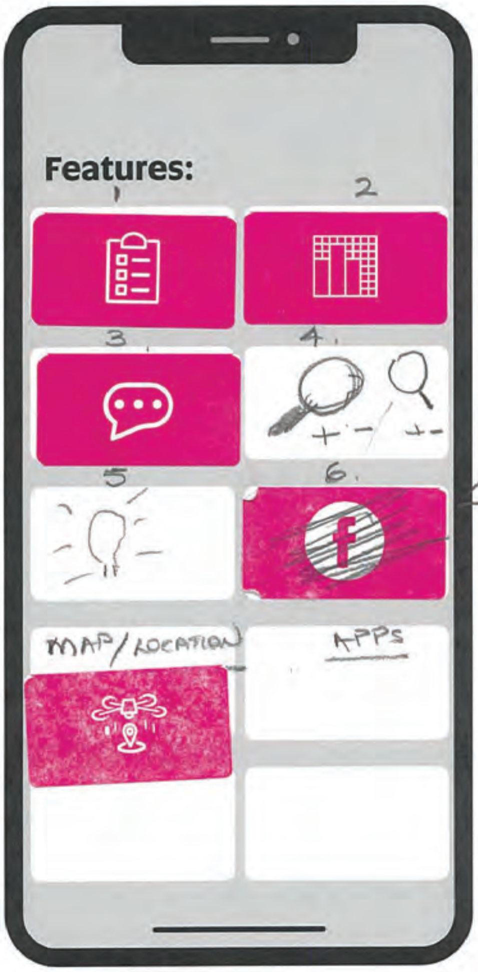

7 MAP/LOCATION
to determine services, walking trails etc. for health + wellbeing around my space.

3. Comment
Opportunity for individuals to comment on articles, opinion pieces/commentary.

8 Apps
– Like the symbol in App Store Apps

4. Searchable Database
of articles, providers of products + services) etc. etc. with indicative costs, council, MAG etc subsidies

5. Tips + Tricks/ Other People's Stories. (from peers who have lived experience)

9 Chat Room/or Facebook Invite Only Group. with an Admin.

AT?

A Day in the life...

Using a self-assessment tool

future proofing your ~~same~~ house.
~~for auditing your house~~

the design clinic

How did you hear about the tool?

from friends & recommended by COTA

Where did you go to get the tool?

A website.

How did you decide to use the tool?

~~It was Word of mouth~~

Going back in time...

Start Here

Thinking back to the features you would like to see in a self-assessment tool, what is the first thing you might do with the tool?

I want to know if our stairs are too dangerous to easily make safe.

~~I do a room-by-room audit of safety fe~~

What happens next?

I answer a ~~[illegible]~~ series of questions about the design of the stairs and how they are used.

And after that?

I get some suggestions for action / next steps

What is the last thing that happens when using the tool?

What do you do after using the tool? and when does this happen?

A few years later...

When faced with a decision about your home a few years later, what do you do?

What happens next?

And then...?

A Day in the life...

Using a self-assessment tool

Start Here
Thinking back to the features you would like to see in a self-assessment tool, what is the first thing you might do with the tool?

What I'm looking for? Age proofing my home
- articles to save
- Tools
- checklist

Saw advertised on Facebook
- "Staying in Place"

Going back in time...

How did you hear about the tool?

→ Facebook ?
→ Uni newsletters !?
→ Local Council newsletter

Where did you go to get the tool?

App Store

How did you decide to use the tool?

- (1) Interest in topic
- (2) Looking for links and tips
- (3) Preventative tips

NOT wanting to end up in aged care

What happens next?

Go to the App Store
- download.

And after that?

- Register – details location etc.
→ Look at how comprehensive menu is
→ Look at who has developed it. (credibility)
→ Ease of use - intuitive
→ Can I add to it ??

What is the last thing that happens when using the tool?

→ Sign off ??
- Searches can be saved & stored ?? so easily accessible.

What do you do after using the tool? and when does this happen?

→ Use it as an ongoing reference tool / Research
→ Timeframe – ?
- ongoing ?
- as needs basis
→ Keep adding to it?

A few years later...

When faced with a decision about your home a few years later, what do you do?

→ Look back at information resources

P.S. Are they still relevant? Will I remember !!

What happens next?

—

And then...?

A Day in the life...

Using a self-assessment tool

the design clinic

How did you hear about the tool?

Where did you go to get the tool?

APP STORE ONLY

NO SIDELOADING (SECURITY REASONS)

How did you decide to use the tool?

AWARE OF NEED TO MAKE LIFESTYLE DECISIONS + CHANGES APPROPRIATE TO AGE.

Going back in time...

Start Here

Thinking back to the features you would like to see in a self-assessment tool, what is the first thing you might do with the tool?

(1) ENTER PROFILE

What happens next?

(2) SELECT WHAT TO CONSIDER FROM OPTIONS OFFERED

And after that?

WORK THROUGH OPTIONS IN PREFERRED ORDER, STORING ANY PROGRESS (FROM PROMPTS OR FOLLOWED LINKS)

What is the last thing that happens when using the tool?

IT PRODUCES AN ACTION CHECKLIST FROM STORED ITEMS. THIS REMAINS DYNAMIC... IS NOT FINAL.

What do you do after using the tool? and when does this happen?

WORK THROUGH CHECKLIST, AS TIME, BUDGET + INCLINATION PERMIT,

A few years later...

When faced with a decision about your home a few years later, what do you do?

REVIEW APP RESULTS, PERHAPS AMEND THEM

What happens next?

MAKE FURTHER CHANGES OR DECIDE ON NEW APPROACH. E.G. TO RELOCATE.

And then...?

GROW OLD + DIE.

A Day in the life...

Using a self-assessment tool

the design clinic

How did you hear about the tool?

Friend or Foe!
FB or ~~Gaggs~~ Google!

Where did you go to get the tool?

App Store

How did you decide to use the tool?

recomendation usually from a friend or foe

Going back in time...

Start Here

Thinking back to the features you would like to see in a self-assessment tool, what is the first thing you might do with the tool?

Download App called My App OR ME!

What happens next?

Set up face Recognition
photo download

And after that?

Set up all Personal information
Bank details
Contacts
Calendar
Medical details
Plants

What is the last thing that happens when using the tool?

Log out

What do you do after using the tool? and when does this happen?

Close the app and all responding details.

A few years later...

When faced with a decision about your home a few years later, what do you do?

retrieve information from icloud that is applicable

What happens next?

informed decision can be made

And then...?

Proof of decision made years earlier

A Day in the life...
Using a self-assessment tool

the design clinic

How did you hear about the tool?
Aging & Govt websites or eminents.

Where did you go to get the tool?
App Store

How did you decide to use the tool?
Noticed life was getting harder to do things you previously could do very easily.

Going back in time...

Start Here
Thinking back to the features you would like to see in a self-assessment tool, what is the first thing you might do with the tool?

Input my "current situation"
Home layout.
Cooking.
Technology
Health

What happens next?
Identify areas where my capabilities are declining and opportunities to learn new skills.

And after that?
Research solutions to abate ~~[illegible]~~ declining capabilities.
Discover new learning opportunities.

What is the last thing that happens when using the tool?
Input what actions you will carry out and then log when you have done them.
Update "Current Situation"

What do you do after using the tool? and when does this happen?
Start the process again from the "new" Current Situation identifying capability decline &/or new learning opportunities

A few years later...

When faced with a decision about your home a few years later, what do you do?
Review where you have come from, ~~[illegible]~~ acknowledge your changing life circumstances, make changes to make life easier.

What happens next?
Assess whether the changes made have actually improved your life style.

And then...?

A Day in the life...

Using a self-assessment tool

"The app that ages with you" ~ adaptable as time goes by.

the design clinic

How did you hear about the tool?

Council for the Ageing & Friend's recommendation
ABC or SBS

Where did you go to get the tool?

Download from app store
Download from Google or trusted website.

How did you decide to use the tool?

* Reviews online or friend's recommendation.
* Free or free trial (full, premium).

No ads. !

promise of personalised app.

Going back in time...

Start Here

Thinking back to the features you would like to see in a self-assessment tool, what is the first thing you might do with the tool?

CHOOSE from a MENU of options
OR
ANSWER a QUESTION.
Press / click on your choice....

What happens next?

App takes me to a screen with a picture and summary of the area of the home or specific problem. Then asks more questions/options. (Can also navigate back to start).

And after that?

Select answers, app provides possible solutions &/or links to other information, or more questions

What is the last thing that happens when using the tool?

I choose what to do and .. app records my decision & relevant info e.g. how to implement, cost, contacts etc.

What do you do after using the tool? and when does this happen?

Opportunity to read or print a summary of the solution (s).

A few years later...

When faced with a decision about your home a few years later, what do you do?

Log of previous solutions kept.
I can now update with or without new info with deleting previous info.

What happens next?

→ Fix the problem.
+ → Offer OTHER solutions?

And then...?

Opportunity to give feedback which can allow developers to update the app design &/or content for "continuous improvement".

A Day in the life...

Using a self-assessment tool

the design clinic

How did you hear about the tool?

Where did you go to get the tool?

How did you decide to use the tool?

Going back in time...

Start Here

Thinking back to the features you would like to see in a self-assessment tool, what is the first thing you might do with the tool?

Store all important + relevant information, files etc. easy to locate
Floorplans + all home features.
All in 'one shop' info with important reminders set
Fitness - Meditation

What happens next?

App guides to the relevant info needed, e.g. will find the document I need
Update itself for relevant reminders

Encourages me to be active or [illegible] I need to meditate

And after that?

Add more necessary tools I need, update

What is the last thing that happens when using the tool?

What do you do after using the tool? and when does this happen?

A few years later...

When faced with a decision about your home a few years later, what do you do?

What happens next?

And then...?

A Day in the life...
Using a self-assessment tool

the design clinic

How did you hear about the tool?

- App Store
- Word of mouth
- other groups I am a member of. eg COTA
- local library
- Radio National.
- OTS Morning TV, Programs.

Where did you go to get the tool?

App Store - Apple

How did you decide to use the tool?

Download. Check it out. Either decide to use it + trial it or Delete it

Going back in time...

Start Here
Thinking back to the features you would like to see in a self-assessment tool, what is the first thing you might do with the tool?

Sign in. Go to settings & set it up. to meet my preferences + security settings. + notifications

What happens next?

Browse the full functionality of the app.

And after that?

Select something on the menu or something featured to have a look/read/exploration, eg. on year(s) of home for optimising a home for ageing well

What is the last thing that happens when using the tool?

Scroll to the top + close the app.

What do you do after using the tool? and when does this happen?

It depends on how up to date it is. If it adds new content daily/weekly I will go back daily. Particularly if I get notifications

A few years later...

When faced with a decision about your home a few years later, what do you do?

A few years later? Too much time has elapsed. I would tend to use an app like This - been there, I was considering "changes" That having been said I might review, seek advice or providers,

What happens next?

Seeking quotes.

And then...?

Redesign,

A Day in the life...
Using a self-assessment tool

the design clinic

How did you hear about the tool?

Radio National.

Where did you go to get the tool?

Google or Play Store

How did you decide to use the tool?

check it out to see whats possible.

Going back in time...

Start Here

Thinking back to the features you would like to see in a self-assessment tool, what is the first thing you might do with the tool?

a check of house circulation & layout

What happens next?

identify issues & how they can be improved

Record somehow

And after that?

file away the information

What is the last thing that happens when using the tool?

What do you do after using the tool? and when does this happen?

A few years later...

When faced with a decision about your home a few years later, what do you do?

assess it for a potential decline in our mobility.

Is house still user friendly

What happens next?

move or modify/renovate

refer to App/tool

And then...?

make a decision based on whats available in market

how hard to change.

A Day in the life...
Using a self-assessment tool

the design clinic

How did you hear about the tool?
APP STORE
FRIENDS
NEWSLETTERS.

Where did you go to get the tool?
APP STORE

How did you decide to use the tool?
- FROM OTHER USERS.
-

Going back in time...

Start Here
Thinking back to the features you would like to see in a self-assessment tool, what is the first thing you might do with the tool?

"PLAY AROUND" WITH IT TO KNOW HOW TO NAVIGATE THROUGH THE TOOL, AND UNDERSTAND ALL THE FEATURES. ALSO TO TAILORISE IT (IF POSSIBLE) TO MY PREFERENCES.

What happens next?
- USE IT FOR A SPECIFIC PURPOSE – WOULD AIM FOR SOMETHING "SMALL" TO START WITH – E.G. FOR HOME IMPROVEMENT, A MINOR ROOM MODIFICATION.
- WOULD WORK THROUGH WITH A FAMILY MEMBER IF NEED BE.

And after that?
WHEN CONFIDENT, USE IT FOR MORE DETAILED PURPOSES, AND ANY ADVANCED FEATURES.
~~WILL MAY ALSO~~

What is the last thing that happens when using the tool?
I MAKE A SELF ASSESSMENT ON WHETHER I FEEL IT'S WORTH USING AGAIN.

What do you do after using the tool? and when does this happen?
(ASSUMING TOOL A GOOD).
- SAVE OFF FOR FUTURE USE
- MAY RECOMMEND TO OTHERS

A few years later...

When faced with a decision about your home a few years later, what do you do?
① Look to see if any other Apps may help me.
- Do I even need an app?

What happens next?

And then...?

A Day in the life...
Using a self-assessment tool

the design clinic

How did you hear about the tool?

~~Ad Advertiser~~
Adverts - TV/FB/Newspaper
Radio/junk mail
contact from agencies
I have involvement with
GP etc.

Where did you go to get the tool?

App store / play store.

How did you decide to use the tool?

Aware that I am ageing
things are becoming harder
looking for solutions

Going back in time...

Start Here
Thinking back to the features you would like to see in a self-assessment tool, what is the first thing you might do with the tool?

Look at all the features,
settings - font size, colours etc
select menu - select room to
assess - set speaker on
- checklist appears

What happens next?

Go into room, go through
checklist - yes, no, maybe, N/A
- dependent on answer other
options appear - do you want
more info now or later (apply
to all selections) - now - suggestions
for changes, links, labels
- options appear as a downloadable
list

And after that?

prioritise actions to take
set timeframes
Resources required
expected cost

What is the last thing that happens when using the tool?

calendar of changes to
make c̄ dates / costings
Downloadable / portable
- option to engage specialist
tradespeople (recommended/
trustworthy) - community option
etc.

What do you do after using the tool? and when does this happen?

start actioning required
change in line c̄ calendar

A few years later...

When faced with a decision about your home a few years later, what do you do?

Reuse the app to see if
further changes / updated
solution etc

What happens next?

Implement

And then...?

Round & Round & Round
& Round!

A Day in the life...
Using a self-assessment tool

the design clinic

How did you hear about the tool?
Refered by professional person/body (Physio, OT, GP, Council)

Where did you go to get the tool?
Directed by them

How did you decide to use the tool?
Recomended
Gut feeling
Free !!!

Going back in time...

Start Here
Thinking back to the features you would like to see in a self-assessment tool, what is the first thing you might do with the tool?

Start a survey of an existing house/garden with the view to make alteration/renovation/improvements

What happens next?
Start questioning what information the app contains

And after that?
Build up a picture of the overall scope of works

What is the last thing that happens when using the tool?
Saves all the information imputed about each designated area

What do you do after using the tool? and when does this happen?
Scope of works sets priorities and indicative cost

A few years later...

When faced with a decision about your home a few years later, what do you do?
Reopen the survey of the designated area, edit and update the survey information.
Re-survey using upto date information
Repeat scope of works etc.
Set reminder to revisit

What happens next?
New scope of works sets priority etc.

And then...?
Do it when $ available
