# Supplementary material for: Self-assessment of the home environment to plan for successful ageing: Report from a digital health co-design workshop
Source: PLOS Digit Health. 2022 Jul 7;1(7):e0000069. doi: 10.1371/journal.pdig.0000069 (PMC9931232; doi:10.1371/journal.pdig.0000069)
Supplement: S3 Appendix — (PDF) [file pdig.0000069.s004.pdf]

Speaker 1:

[crosstalk 00:00:00]. A couple of key points about your discussions

Speaker 2:

And just a reminder, this is when we are sharing our information and the recorder's on.

Speaker 1:

Okay. So we're going to start here and then we'll come over to this table and then we'll finish off with this table here

Speaker 10:

So just a couple of points on the perfect home. I'm sure many of us will agree on these, but our group, particularly most of us liked the idea of a garden. Thought it was essential to have some sort of a garden, a low maintenance, easy to look after accessible garden, but somewhere one could get out into nature. But also to have a home that would give us access to being able to do the things that we need to do and want to do, whether that's just daily activities like cooking and washing, but also pleasure activities. Like for me, that's yoga and dancing to music, stuff like that. So low maintenance, easy care and pleasant.

And the second topic was very interesting. We had a very interesting discussion about that. Enjoyed the challenge of thinking about, do we want a home that's going to challenge us to stay fit? But then what do you do if something happens? You want to be able to, for example, have high coverage, so you're reaching and stretching, or maybe you want stairs so that you're keeping your legs fit, which is what my mom did. But then, what happens if you then can't use those stairs? So we kind of came to a point where we said, we need a house to be adaptable. We need to be able to have some way of changing things around at a different stage of our development or if we have an accident or something like that.

Speaker 1:

Obviously.

Speaker 2:

Yes, anyone else from that group want to add anything?

Speaker 4:

I think, the only other thing I would probably add to that is the access to community and being able to access facilities and keep connected with the community. And I think we generally agreed on this table that we didn't particularly want our community to be all old coaches. We want access to young people as well. So like keeping it mixed and varied. Yeah.

Speaker 1:

Fantastic. We'll move the report across to-

Speaker 2:

Second group.

Speaker 1:

They're labeled as group one on the computer screens.

Speaker 5:

I mean, we talked about some of the things, but then it's also occurred to us that it's not just the design of the home that's important. It's where the home can be, is really critical. So somewhere where you can go out and walk by yourself and it's safe to do that. And it's nice to do that. So somewhere where you've got shade and some on the streets and close enough where you've got somewhere, you can go and get milk in a five minute walk, even on a frame or something, so that it allows you to actually keep yourself active. So it's not just the design of the house inside it, but it's actually where the house is situated. Because one of the other things is the garden again, but it's a garden that gets sun, particularly in winter. If you're shaded by a big apartment block or something and you never see the sun, what's the point of having the garden because it's too cold to go out to.

So there's a whole range of things like this that I think that we thought of and definitely somewhere where you can have friends in and a house where you can cook and you can host people to come. Maybe a spare room where people can come and stay as people go, whether they want to by then, who knows, but that kind of thing. And somewhere where there are the obvious things like being safe and all those kind of things as well, I'm comfortable, is possible. Something to be warm in winter and cool in summer. Those kind of things. It's the design of the house itself, maybe as much as what's specifically in the house.

And how can a home keep you fit and healthy? We found this one a bit more difficult to kind of get a handle on, but things like a house that encourages you to walk around and move. So it's not where everything, the old fashions [inaudible 00:04:51], the old fashioned be. It's terribly last decade where kitchens, you never have to move. You just stand at one spot and do everything, right?

Speaker 2:

Right?

Speaker 5:

Whereas in our next couple of decades, we are going to need kitchens. Now, probably, where you need to walk from one side of the kitchen to the other, to keep yourself moving. There's stuff like that I think are really important. And places that are easy to clean, but possible to clean as well where you can keep doing stuff and keep doing a bit of gardening and keep walking around the place and possibly, do you want stairs or not? Stairs can keep you really active, but they can also suddenly become a danger.

Speaker 2:

Often that.

Speaker 5:

So it's that kind of, again, both that flexibility about you can change things, if you can adapt things as you go.

Speaker 2:

So we were saying, if you build a staircase...

Speaker 5:

Yeah.

Speaker 2:

Sorry. Just kind of asking, if you look at a buying a place that's got a staircase, but if it's so narrow that you can't put an acorn chair lift to go up for example, then so the design of a house again.

Speaker 5:

Yeah.

Speaker 1:

Okay. Anyone else in that group want to add anything?

Speaker 2:

No, that was covered. Very nice.

Speaker 1:

Yeah.

Speaker 2:

Yes.

Speaker 3:

Okay. We'll move our recorder across to our final group here for last thought before copy.

Speaker 6:

Okay. So imagining ourselves in that 85-95 age group, thinking what a perfect home would allow you to do, the main thing for us was that it would enable you to do whatever you do now, but safely.

Speaker 1:

Yeah.

Speaker 6:

So it was about, again, access to outdoor spaces, not being slippery, moving from room to room easily, taking into account that you might have walkers or wheelchairs. And we also thought an ability to close off open spaces, Because there may be situations where you don't want it to be totally open. So that was kind of where we got to on that one. And then with the, how can a home keep you fit and healthy? We felt the most important thing was that no one size fits all. People have different comorbidities, which will impact how a house is. So it needs to be adaptable for each individual and it needs to be whatever solutions. It needs to be that the solution fits the individual, not that the individual has to fit the solution.

Speaker 1:

Fantastic. Anyone else on that table want to add those points?

Speaker 7:

No.

Speaker 1:

No? Okay.

Speaker 3:

That's a nice summary.

Speaker 1:

All right. Summary. And I think it's really nice to see some nodding around the room, that most of us are kind of getting on to the same page around these key principles of adaptable and flexible. And we had a [inaudible 00:07:53]-

Speaker 1:

Pull up your images, so everyone can see which ones they were. Now, all three groups have the same 99 images. So, you should recognize the ones that you are seeing. {de-identified}, do you want to just hold them up a bit closer so everyone can see.

Speaker 2:

All right, well... the first one was... I think with the hot water bottle was sort of symbolizing sort of security and warmth down the [inaudible 00:00:50]

Speaker 1:

Security and warmth was the hot water bottle.

Speaker 2:

The second one was symbolizing about technology as we're moving forward, that it would be important for homes to have and we have to adapt to technology. The third one was around gatherings, people coming so around sharing your home with other people [inaudible 00:01:12]. And the last one, the painting was about creativity and that for mental wellbeing, to be able to within a home, be able to do, to have opportunity for doing creative things for.

Speaker 3:

Okay. So we were... Because one of our team members is indecisive... We picked up on that... We've got eight, but actually pretty much very similar to those. So the nature piece, access to nature and light is the first one. Spaces, they can be adaptable. So there can be comfortable spaces, but it can also be opened up... That's inside the house, outside the house, inside the house. And these are more aspirational ideas around the support system. So the house has to be within a system that does support, hence the sculpture and the tree, and another aspiration is that it is aspirational. It's inspirational. So we want spaces that make us be the best version of ourselves.

Speaker 1:

Fantastic. And {de-identified}, if we could just pass the Dictaphone across to number two... And who will like to come up and tell us about your images here?

Speaker 4:

Okay. So we managed to get it down to five. We're not quite as indecisive. All right, first one is warmth and light. And the reason for it is it's a [inaudible 00:02:48] comfort, optimism, wellbeing, art, and creativity, much the same as over there... We are creative beings, it's good for the soul. Freedom and fresh air, and that's just because that's just what it is. Good food and good friends, culture, interaction, humor, laughter that sort of thing. And then the last one, but not least of course is learning and relaxation. And just because if we don't do it, then we lose it. That's it, stop and you're dead.

Speaker 1:

Fantastic. And our final group, number one, who have managed to get it down to four, again our superstar group over here.

Speaker 5:

[crosstalk 00:03:38] These are our images. The first one is sweet and sour... Fresh, colorful, tasty, flavorful, diverse. Love and community is the second one, and different age groups was important too. Keeping connected with the whole gamut of people and loneliness kills if you're not. This one, the view from my window, it's about a sense of space and having a visitor, you don't have to have it all in your land, but you've got that, a sense of space and the importance of not being hemmed in. And the last one is transience, life goes on without you, in the end nothing matters, but it also smelled green, I thought.

Speaker 1:

Fantastic, so three very different combinations there.

Moderator:

So would someone like to kick us off over on this table with what we've picked and a little bit about why?

Speaker 2:

I don't think I will. I think it's fairly straightforward.

Speaker 3:

I have to say, I probably selected things that aren't necessarily so much about aging as just things that need to generally be done before I get too old to be able to do it. And a lot of it is the hard and the medium is based on the negotiation with my partner, not so much around the money, but it's also space. We're in a small house on a small block and trying to perhaps install another toilet somewhere isn't that easy because you almost have to knock a wall out just to do that sort of thing. So it's just generally trying to make it a bit more friendly, I guess, to two people, in a way. Doesn't make sense. It's a small house. We've come from large houses into a small house. And as you try and negotiate where to put things and what has to go, it's always a bit tricky. That's not necessarily about aging, so that's not very helpful. You can put pause on that.

Moderator:

Yeah. You want to [crosstalk 00:01:27]?

Speaker 4:

Yeah. Well, I think really my choices of whether it was hard, medium, or medium, I didn't actually say anything was easy, is mainly based on cost, because that's kind of at the forefront of my mind, what things are going to cost. And some of the things are things that to be done, and some of the things are specifically about what needs to be done in the next 10 years because of aging considerations. So it's a bit of a mixture of things. But it's really good it's made me think about those things.

Moderator:

Now, {de-identified}, I'm interested that you finally said there's two things that need changing in your house.

{de-identified}:

I will say, to kick it all off, we have spent a lot of money and a lot of time in the last decade doing things like handrails and nonslip floors, everything like that. Having gone through {de-identified} you start getting very good at that sort of stuff. The hardest thing for me was natural breezes. We're actually in a little bit of a shadow, so we don't get any rain, well, hardly any rain. We get hardly any breezes or anything like that. So that's the one that's hard and couldn't be done.

Moderator:

Okay. That's brilliant. I think we might-

Moderator:

So I might move our recorder over to {de-identified}, do you want to report any of those points or share yours?

{de-identified}:

Okay. So being realistic, our back garden has some things that really need doing. It needs retaining walls. Thinking as we get older, it does have a number of different levels, so steps and slopes and things like that. I put that as hard because that's mega dollars, which we don't have, and neither of us are physically able to do these things anymore.

The second part of that is we actually have a very large garden, which is too big to look after, but having said that, neither of us want to get rid of it. So we're just going to have to live with that one. A couple of easy things. Yes, we do have handrails, et cetera, but I think there are some other areas where we need handrails. We have a very long hallway. He says the floor's not slippery. I think it will be as we age, taking into account watching what my mother falls over on. So I think handrails down there is going to become important.

Then again, the other hard one is going to be the general renovations, because again, that's going to be the dollars, which is painting, replacing a couple of ceilings, those sorts of things. They, it's all the money. And lastly, we need to do a big declutter, which is storage spaces. And I've said that's medium, because unfortunately, a lot of that hangs on my mother’s health.

Moderator:

Thank you for sharing. Anyone else on this table want a go at telling us their story of what they've chosen? Yeah, please go ahead.

Speaker 7:

Okay. A lot of the things I had already put into place, thinking about... Having cared for my late mum for six years before she passed away, that forewarned me, I guess, of some of the things that I would need to consider. And so, as I was working, I guess, towards the sort of retirement age, I considered things like the reverse cycle air conditioning, a whole lot of other things that were already mentioned there. What I haven't addressed at the moment has been the handrails. And I guess I've held off on those simply, I guess, ego, probably, sort of coming to an acceptance that I really need them. So that's one issue.

They're pretty easy to install. What I thought were probably another thing that was easy to address was the furniture that I had in my bedroom. And I thought that as I became less mobile, I would probably need to declutter a bit. But that, again, was also pretty easy. Some of the things which I considered needing to be done were changing the shower to allow for, I guess,

a chair within the shower, someone else to wash you, should you get to a point where you can no longer wash yourself. Changing the shower head to a handheld so that someone else could wash you.

That would probably require the actual frame of the shower to be increased in size, which may then impact on plumbing and et cetera. So that could be a medium to hard one, and obviously possibly a costly one. The other thing which I think could be quite difficult to do now would be changing the doorways, which are standard size. And they don't allow for walkers, nor do they really easily... Except for hallway, which is quite wide. The other doors don't allow for wheelchair access either. So should you need that in the future, that could be quite hard to do now.

Oh, one other thing, peace and quiet. Peace and quiet was not for me within the home. It was from all of the neighbors around you. And whilst we talk about intergenerational living, it can be very difficult when you are elderly or frail or not well, and you have screaming kids on the one side

Speaker 4:

[inaudible 00:08:38], like to share as well?

Speaker 8:

Yeah. A couple of the other ones I had down, and it's interesting because I think it's been touched on here. My wife and I actually sat down thinking about this about a month ago, because we decided we want to stay in our current house for as long as we can. So some of the things were, we've got a large garden and my wife loves gardening at the moment, but we know as we get older, we're not going to be able to do it. So we need to adapt that garden to be a low maintenance garden, get rid of all the annual cottage stuff and replace them with natives. So that's something we've got down to do over the coming year or two.

The other one, I didn't even think of it. Light switches, I also had down about space between furniture. Because I think in our house, we've got a lot of power points behind furniture at the moment. As we get older, it gets harder to get to when you need to adapt them. So we need to think about moving furniture around and maybe getting some of those light switches up high rather than down, six inches off the floor, which they are sort of at the moment. And the hand rails as well, and indoor temperature. Our heating's only in one part of the house. It'd be expensive, but ideally we should get central heating to ideally go through the whole house for winter time, so we're not just sitting in the lounge room at night.

Speaker 4:

Good point.

Moderator:

{de-identified}, did you want to say anything?

Speaker 8:

Yeah, more or less to say we probably move. It would be so much [inaudible 00:10:02] this house. So I'm looking forward to it already, because I love gardening. And the plot where we are living, it's absolutely beautiful and fantastic. It would be really terrible to leave this area, but the house doesn't suit us now and it will not suit us in the future. So it's really something where I think it's a special house and you need special people to live in it. So that's all what I can say. So there are a lot of [inaudible 00:10:34] lots to do. And if I don't want to do the gardening anymore, we probably have to move.

Moderator:

And {de-identified}, did you want to share your reflection on this activity as well?

{De-identified}:

Yeah. I'll have a go at this. I started laying the cards out, and to me, I struggled with the whole concept that I need to change anything in my life, and struggling with the idea that I will more than like to have to do this at some point. So I think there's an interesting thing around denial or the resistance to recognize change.

Speaker 7:

Barriers to change.

{De-identified}:

And then as I looked at it, I started seeing yeah, those tiles are probably really slippery in the bathroom, and [inaudible 00:11:18] the stairs might become an issue, but there is definitely an initial resistance to saying, this is the whole thing, that it's not going to happen to me. Age isn't going to happen to me. So I think conceptually, it's quite a challenging set of questions, I think. That was my observation anyway.

Moderator:

Come to our last table [crosstalk 00:11:39]. Oh, sorry. Thank you. [inaudible 00:11:42] bring that one. I don't even need to be here. This is great. Anyone want to jump in and go first on this table?

{De-identified}:

Okay. I'll stand up, because it's better, I think. One of my low priorities, but you mentioned it, is bathroom handrails. I've lived with those {de-identified} and they're bloody wonderful, even if you don't need them. You want to wash your leg, you grab the... So don't be afraid for ego reasons to get-

Speaker 12:

They just look ugly. If they had better designs to them, I'd feel better.

{De-identified}:

Well, I reckon all houses should just have them and then nobody would be fussed about it. I spend a lot of time in front of the computer, in a chair, and I really need to make that comfortable, because I spend hours and hours every day there, writing and stuff, and reading. Space around in between furniture. I live in a tiny little apartment and the landlord supplied the furniture, or most of it. And it's got this huge coffee table that takes up most of the living area, and that needs to go. Also, we haven't mentioned, but it's got very sharp corners. Now, if you have a fall, you can be killed. Things should have round corners and preferably softer edges.

Anyway, slippery floor. The bathroom, when the floor gets wet, that's a skating rink. So I need to fix that. Connection to the neighbors. That's just like a matter of doing it. I tend to isolate myself and I shouldn't. I mean, nothing could be easier than just introducing yourself, but I don't do it. And it's something I... As you get older, I think it's nice to have someone you can have a cup of tea and a chat with. Natural breezes. All I have to do is open a window, but I don't do it often enough. And that's really it, so there you go.

Speaker 11:

Fantastic. Thank you

{De-identified}:

I just wanted to talk about the large staircase and narrow doorways. Hopefully, I'm not going to need them, to change them at all, but more than a handrail down the staircase would do it, but who knows? And if it's narrow doorways... We've kind of bought a house five years ago, and with the understanding it's a 10 year project. I turned 70 this year. So by the time I'm 80, we kind of see that we might have to move out anyway. And I think the most important thing is to just have thought about it and have a plan, some sort of plan going forward, otherwise you end up... As we were saying, I don't think the age care facilities realize what's coming-

Speaker 12:

With us. Us lot, us lot.

{De-identified}:

... with our generation. I think there's going to be real trouble.

Speaker 12:

Yes. Good. About time.

{De-identified}:

We're going to have a whole lot of very bolshy old people.

{De-identified}:

It's going to be trouble, anyway. They're going to have to adapt really fast anyway. So who knows in 10 years time or five years time now, 10 years time, what's going to be happening? But handrails, I think, would probably solve a lot of issues later on.

Speaker 12:

I agree. Can I just comment? As I said before, I think one of the issues, some of it's, things like handrails, around egos. Every time I see them, they're ugly, as a design thing.. But good design doesn't have to be expensive, and useful things don't have to be ugly. That's my bit. But for old people, it's almost like, "Oh, it doesn't matter. Just give them something. It's fine." Always, again, that's an issue for most of Australian houses. They're not built for anyone who's on any age, who's got to go on a frame or anything like that, and that does mean... My place is not so bad because it's fibro cottage, so structurally it's not hard.

Natural light. That's a bit of an issue for me, not in the morning, but in the afternoon. That might just be skylights in a few more places. Big thing for me is terraced garden. And I'm currently using somebody who comes twice a year. If I want to stay where I am for a few more years, I'm going to need to spend more on someone doing some gardening. Or as I was just saying to {de-identified}, age proof the garden a bit more. There's plenty of agapanthus I can share up the back garden. So, it's that sort of thing.

I think one of the other issues is just the general maintenance and renovations and whether they're cheaper if you stay where you are, because you've still got to buy and sell somewhere else. Who knows? But mostly, I have completely age proofed the bathroom already, so that's done, and most of the rest of it's quite easy, and the hard bits are all about money.

Speaker 14:

This was really hard for me, because we've just moved into a brand new apartment. This was all done due to the fact that my husband, this gentleman, broke his hip, broke his leg, back surgery, arm surgery, shoulder surgery. So we had to already do this. And the only thing that we would really do now is, within I suppose the next five years, would be the second bedroom would become one of our bedrooms, because of his age, and the bathroom handles. And the peace and quiet.

Speaker 12:

Can I just throw one more thing in? Having left one house because of neighbors from hell, everyone talks about making friends with your neighbors. A couple of other people have mentioned, this is not a given. I know another friend who's about to leave because of the neighbors. There's this common thing about being friends with your neighbors, but they might not be people you'd ever want to even pass the time of day with.

Speaker 15:

That's true. Yeah.

Speaker 12:

And that is assumption that's quite incorrect often.

Speaker 11:

And conversely, the issue of people shouldn't have to be forced out of their homes because of neighbors who are neighbors from hell, but nothing seems to be done about them anybody.

Speaker 12:

No, that's right.

Moderator:

Excellent.

Speaker 11:

Can I also throw one other in?

Moderator:

Yep.

Speaker 11:

I guess as we're talking about future proofing our homes, we haven't considered future proofing our homes so that as technology develops we are actually ready for it. I can see, for example, in our place, we've had to put security cameras in. We've had to do all sorts of things just to improve our level of technology use. And I'm pretty tech savvy, so I'm aware of what I need to do, but a lot of people may not be, and it's moving really, really quickly.

Speaker 16:

Actually, I've something I want to say too, if that's all right.

Moderator:

Yeah.

Speaker 16:

I have noticed with my own relatives that there's a resistance to leaving the home, and this determination to cling to it and adapt to it, which is not always really all that successful or durable, as an adaptation. But then when they have to move, they're happy as Larry. "Oh, this place is terrific." So I would like to see a removal of barriers to moving, and one of those is the cost of moving. When you buy and sell a house, the government takes 4% off your transaction or something. Then there's the real estate agent. Then there's the physical cost of moving, which is huge. If we lubricated the process of people moving to appropriate accommodation, there'd be less to spend on struggling to make the accommodation they've got suit them.

Moderator:

This is absolutely fantastic

Speaker 1:

Sorry. Just a warning that the dictaphone is on. There is no pressure to say anything while the recording is on. We will tell you the moment it is turned off as well. But did anyone on this want to share any key insights or things that you think it really should be doing?

Speaker 3:

Yeah, we haven't discussed it as a general thing, but I think the idea of using the app or whatever, an app as a storage space to keep the research you're doing. That the app is guiding you through and trying to fix things. It's hoping... Hopefully the app will help you come up with solutions for problems that are there, that you're not even aware of currently. And therefore you're going to need somewhere to keep things and to push you and to guide you into finding out how to solve it and where to get stuff, which the app can't solve all those things, but it can actually point you in the right direction and give you the kind of terms for Google to put into a search engine or ideas for going to the websites and looking at online hardware shops and see what's available and all that kind of stuff. And somewhere to bring all that stuff together again.

Speaker 4:

And store it.

Speaker 3:

And the other... And this is... That was a general kind of conversation, but my... I would also like the app to be able to compare.

I want to see where I fit in with general other people too. And so I would like some kind of graphic or graph to come back to me, showing how my responses fit into the general kind of group of old people around the place.

Speaker 1:

And do you want to know who those other people are or anonymous?

Speaker 2:

Anonymous. Anonymous.

Speaker 3:

It could be other users of the app.

Speaker 4:

Yeah.

Speaker 3:

It could just be as simple as what? Simple as... as straightforward as that.

Speaker 1:

What if it was your friends? Would you want to compare with?

Speaker 3:

No.

Speaker 3:

Spent hours talking about getting all. You don't need to have it anymore.

Speaker 2:

I see it as layered. It's a hub, if you like. And there are the... As {de-identified} said, that the questions around the pointers to lead you down key pathways, but I would love to have something again that would store things and bring material together. I think it needs to be absolutely idiot proof in terms of usability. And I'm speaking for myself here, lest anyone be offended, that there is that scope to be able to do that. And there will be some standard pointers perhaps, but these are key sites that are really useful to look at. And there might even be a little news box that says, "This is a new site that's come up about like..." But I come back to money, money, money to keep all this going and up to date.

Speaker 6:

Mine, I had totally different idea. Mine was to have everything in there that could control my life and help me not forget things. Shopping list, ambulance, medical access to the web, payment of bills, banking, car service, which I forgot last week.

Speaker 4:

Oh.

Speaker 6:

Reminders to call my friends, which I say I'm going to do and then they ring me back and, "You didn't ring me." Filing of important documents that I need, that just seem to get lost by the time I get to June 30th to want to do them again. And a calendar, travel itinerary, passports, all the things you need to travel with.

Speaker 5:

You need a personal assistant.

Speaker 1:

That's no, that's great. And I think a lot of those are really interesting things, particularly it seems to relate to that story, everything so actually know to come back and find it again as well. I'm going to reminders because yeah. My car reminded me the other day. It needs to be serviced and it was very handy cause I had completely forgotten. Now I come across to this. So would anyone want to jump in and share any key features or insights or things that they feel as though it just really should do this based on what you've heard or some other ideas is at all?

Speaker 7:

I always will. I thought one of the real priority on it was to have some sort of settings that you could completely customize the site to you. Whether that's your font size, your colors, whether it plays music while you're working with it. But something that may accept yours, personal.

A menu that allows you to select either rooms or equipment and then sub menus. What do you want to do with that information? Do you want to look at suggestions? Do you want to make some selections of things you think might work? What are comparable costs around all of those, a home key so that you get back to the start when you're lost and confused, always important. And the other thing that I really thought was a speaker, so that when you're actually in a room and you're looking around that the speaker's saying to you now have a look at the space between your chairs or the tables behind minutes. Well, but so that you, but you've gone into the room. That's what you said. You want to do. I want to assess this room. Yeah. And it's asking you that it's talking to, you're not trying to read it and look at the same time, which kind of stops you falling over the coffee table or the dog, whichever comes first, so that's kind of where I wanted to.

Speaker 1:

Excellent. Any other features on that table?

Speaker 8:

One of the biggest things about an icon based system is what you're grounding in icons is. This is very Android oriented. If you are a diseased or iPad user, all the icons are different. So what I thought was that if you hovered over a particular icon, as in, like you can put a label on hyper text, you can put a label on when you hovering over the house and it actually then comes and says whole or comes and says profile, but in big enough writing so that you're not trying to read right there.

Speaker 1:

Yeah. Excellent. Yeah. I wish I could afford stickers that were holo graphics so that we could have multiple things on there, but any other big it's from this table?

Speaker 9:

One thing that I didn't actually can't say that I put on there, but was thinking about was the idea of a forum, which in itself can be a real problem because you get a lot of idiots on there as well. And the information isn't checked, but it's also quite useful. And I don't know how you monitor it, but it's a useful thing. If you are trying to find a good Sparky that understands where PowerPoints need to be located and things like that you can actually say, well, I live in the south and these guys were pretty good. You know, that sort of thing could be quite useful.

Speaker 1:

Kind of Recommendations.

Speaker 9:

Yeah. So it sort of localizes it a bit more to the area. But I also understand the issues around forums that.

Speaker 10:

And Adding to that is I've got one of the dropdown menus is an indicative cost. So that, for instance, if I'm got to put on a handrail and drill through some tile, you need X, Y, and Z. So I'm going to ask a plumber to do, he says, oh yeah, I'll come out for $300 and you go, oh, hang on a minute. This is only going to take you 15 minutes. So it's that sort of, it should only cost about one hours labor, two hours labor an hour and a half labor.

Speaker 11:

Yeah. And also I think attached to that is kind of, or, or information about what supports are available in a local area like councils people and plans that local councils have and stuff to subsidize some of these, these things. We just don't know otherwise.

Speaker 1:

Is there anything else on this table?

Speaker 12:

I just, I mean, I think it will be covered, but the ability somewhere to link for further information as well or detailed information just within the app, but provide me the links and it could be DIY videos. It could be more detailed sides.

Speaker 6:

Function that actually shoots you off to other areas.

Speaker 1:

Actually with what you need to be connected with. Come know if I last table the front here and want to share on these ones, I'm really looking forward to looking into this one into detail. I needed a bigger sheet of paper.

Speaker 13:

Shall I start? So actually what we did was similar to what you did. And we talked a lot about things that would be helpful in general, around the home and in daily life for an app, but some of the stuff is directly relevant to self assessment. And one of those is that the app should ask you questions so that it can tailor itself its responses. And in fact, what it offers you to your answers. And I've had actually a good example of this. I've just recently downloaded a meditation app called Balance that is currently offering a year free. And they do that by asking you questions about your meditation practice and what your goals are. And then the actual app presents you with a program that's tailored. Now, this probably wouldn't want to be quite so directive, but something like that would be really good.

I think, also {de-identified} and I thought age friendly navigation is really important to have a really clear menu with links that will take you to the information that you want or the area that is relevant to you because it's nothing more frustrating than going onto a website or an app and ending up going somewhere where it's repetitive or it's not useful, or you go round and round in circles like any government apps, government websites, and you end up actually not getting the information that you went looking for in the first place. And sometimes you can't even get back to where you started from to start again. So yeah.

Speaker 13:

User friendly navigation. It should be not just age friendly.

Speaker 14:

I think I've got to say right from the beginning that I took, I only looked at this from the perspective of being a tool, helping me in determine whether my home is suitable for aging. So that's all I did. Yeah. And in doing that, I thought, okay. And they're not in any order. So they're just random things. If I was then looking at my home, be it by room specific or any other way, I would want some checklist of features that either I or some other person has given me to sort of say, okay, in a bedroom, these are some of the things that you might want to consider from a technology perspective. These are some of the things that you might consider. So I could have some sort of checklist, but from on that I was going, okay, so let's just say that I did this checklist, where would I want to go?

Where would this then take me, would I want to have a look at what they say, for example, about handles of doors and cupboards and where would that take me? So had all these questions for myself. I would also want to have access to I've called it latest research or evidence that the Soap called. Well, I would expect that they would be experts in their fields, be it home design, be it technology aided and AI homes and safety and security, et cetera. So I could have a look at that and read it if I was interested and then explore something further if I wanted to. A third was if I did read an article that or something that someone had written, be it an expert, or be it a peer who has got expert lived experience, have I got the opportunity to provide comment and feedback so that there is dialogue between the readers, a searchable database and that searchable database could be site-wide.

So it could be articles, experts, something related to design, whatever I wanted the opportunity to share. So if I found something that was really interesting, and yet I thought that could be of interest to a friend or member of my family or someone else in my network, then I could send it on to them separate to Facebook because I am a Facebook user and I do share on Facebook, but I know that not everybody does. Map location, because before we were having a look at a website, which was where you were able to put in your address, that you showed us. And then that highlighted from where we were located, what, what other things in that area were available to us from walking trails to bike, riding to other services, et cetera. So that could be possibly useful apps.

I'm an app user, my phone's full of them. And I think we're, we are moving into that sort of age of our lives, where they're going to become more dominant. So I'd like to be able to find out about latest apps, which I might not otherwise find out about. Someone said earlier on about a chat room. And then I heard what they said about the fact that they need to be moderated, whether it's a forum or a chat room or a closed Facebook group, some avenue by which people could converse with each other, because it's a lot of texting that goes on all the time, but you know, more detail discussion with each other. And I think the last thing I had was tips and tricks. That's what I called it. But it's the idea of my learning from other people who've already been there and being able to ask questions of them if something was of interest. So those are the things that came to mind for me

Speaker 1:

Fantastic.

Speaker 16:

I just found that I was putting, I started out and I finished up inputting a whole lot of data about myself and the way I lived and all of that sort of thing into the app with the program, whatever you like to call it. But then right at the end, I thought this is a really good opportunity for a development so that all of that information can be used and collated to offer me suggestions on a way forward or a different way of living, sort of making use of the information I've put in there in a way that I might not think of. Similar to the program you showed us that website. Yep. That takes you to expand your horizons if you like what is available, what is possible.

Speaker 1:

Sounds a bit similar to some of the things we were talking about earlier on the first table around that profile on the suggestions of things you might not have thought to look at. Yeah. As you're moving through a home. So I'll just get {de-identified} to pause the recording so we can all speak freely again
